# RFX2 Is a Major Transcriptional Regulator of Spermiogenesis

**DOI:** 10.1371/journal.pgen.1005368

**Published:** 2015-07-10

**Authors:** W. Stephen Kistler, Dominique Baas, Sylvain Lemeille, Marie Paschaki, Queralt Seguin-Estevez, Emmanuèle Barras, Wenli Ma, Jean-Luc Duteyrat, Laurette Morlé, Bénédicte Durand, Walter Reith

**Affiliations:** 1 Department of Chemistry and Biochemistry, University of South Carolina, Columbia, South Carolina, United States of America; 2 Centre de Génétique et de Physiologie Moléculaire et Cellulaire, CNRS UMR 5534, Université Claude Bernard Lyon-1, Villeurbanne, Lyon, France; 3 Department of Pathology and Immunology, University of Geneva Medical School, CMU, Geneva, Switzerland; Cornell University, UNITED STATES

## Abstract

Spermatogenesis consists broadly of three phases: proliferation of diploid germ cells, meiosis, and finally extensive differentiation of the haploid cells into effective delivery vehicles for the paternal genome. Despite detailed characterization of many haploid developmental steps leading to sperm, only fragmentary information exists on the control of gene expression underlying these processes. Here we report that the RFX2 transcription factor is a master regulator of genes required for the haploid phase. A targeted mutation of *Rfx2* was created in mice. *Rfx2^-/-^* mice are perfectly viable but show complete male sterility. Spermatogenesis appears to progress unperturbed through meiosis. However, haploid cells undergo a complete arrest in spermatid development just prior to spermatid elongation. Arrested cells show altered Golgi apparatus organization, leading to a deficit in the generation of a spreading acrosomal cap from proacrosomal vesicles. Arrested cells ultimately merge to form giant multinucleated cells released to the epididymis. Spermatids also completely fail to form the flagellar axoneme. RNA-Seq analysis and ChIP-Seq analysis identified 139 genes directly controlled by RFX2 during spermiogenesis. Gene ontology analysis revealed that genes required for cilium function are specifically enriched in down- and upregulated genes showing that RFX2 allows precise temporal expression of ciliary genes. Several genes required for cell adhesion and cytoskeleton remodeling are also downregulated. Comparison of RFX2-regulated genes with those controlled by other major transcriptional regulators of spermiogenesis showed that each controls independent gene sets. Altogether, these observations show that RFX2 plays a major and specific function in spermiogenesis.

## Author Summary

Failure of spermatogenesis, which is presumed to often result from genetic defects, is a common cause of male sterility. Although numerous genes associated with defects in male spermatogenesis have been identified, numerous cases of genetic male infertility remain unelucidated. We report here that the transcription factor RFX2 is a master regulator of gene expression programs required for progression through the haploid phase of spermatogenesis. Male RFX2-deficient mice are completely sterile. Spermatogenesis progresses through meiosis, but haploid cells undergo a complete block in development just prior to spermatid elongation. Gene expression profiling and ChIP-Seq analysis revealed that RFX2 controls key pathways implicated in cilium/flagellum formation, as well as genes implicated in microtubule and vesicle associated transport. The set of genes activated by RFX2 in spermatids exhibits virtually no overlap with those controlled by other known transcriptional regulators of spermiogenesis, establishing RFX2 as an essential new player in this developmental process. RFX2-deficient mice should therefore represent a valuable new model for deciphering the regulatory networks that direct sperm formation, and thereby contribute to the identification of causes of human male infertility.

## Introduction

Reproductive failure affects 10–15% of couples worldwide, with responsibility distributed about equally between males and females [1,2]. Failure of spermatogenesis is a common cause of male infertility. A large fraction of such cases is believed to result from genetic causes. The advent of forward genetics has led to the identification of over 400 genes associated with male spermatogenic defects [1]. However, the precise etiology of most clinical cases of male infertility remains unknown.

The process of sperm production in the testis is usually described in terms of three phases: the multiplication of diploid spermatogonia, the reduction of chromosome number during meiosis in spermatocytes, and the morphological conversion of round, immotile haploid spermatids into nearly mature sperm by the process of spermiogenesis. The overall process is controlled by master external regulators, including retinoic acid, the pituitary gonadotropins, and testosterone [3]. In contrast, details of the process depend in part on local biochemical communications with closely associated Sertoli cells, and a dynamic gene expression program within the germ cells that involves both transcriptional and post-transcriptional regulation [4,5].

The current study addresses the role of a member of the Regulatory Factor X (RFX) family of transcriptional regulators. Early studies led to description of the X-box as a DNA sequence motif conserved in the promoters of genes encoding MHC class II antigen-presenting proteins [6]. Search for the factor that activates MHC class II genes through this motif led progressively to the identification of a family of RFX transcription factors (TFs) that now numbers 8 members in mammals [7,8] and has its evolutionary origins traced to microorganisms. The most fundamental shared feature is a variant of the winged helix DNA binding domain [9]. In the invertebrate metazoan *C*. *elegans* there is single RFX relative (DAF-19), which controls genes important for cilia development and function [10]. In vertebrates several *Rfx* genes have also been shown to control ciliogenesis (for review see [11,12]). The family can be divided into two groups based on their ability to form homo and heterodimers: RFX 1–4, 6 and 8 are known or predicted to have this property, while RFX5 and 7 do not. This capacity to bind as heterodimers to the same DNA motif makes unambiguous assignment of functional roles to individual RFX proteins difficult when multiple members are present in the same cell.

Mutational analysis has established critical roles for most *Rfx* genes. *Rfx1* deletion in mice was found to be embryonic lethal, in keeping with reports of a wide variety of potential roles [13]. Mouse *Rfx3* is critical for cilia development and function, and its absence leads to a variety of severe defects in left/right body patterning, CNS development and differentiation of endocrine cells in the pancreas [14–16]. Disruption of mouse *Rfx4* causes failure in the development of dorsal midline brain structures. It is also critical for formation of the subcommissural organ [17–19]. *Rfx5* was the object of the original search for the X box regulatory factor, and is strictly required for MHC class II gene expression in humans and mice [20]. Genetic ablation of *Rfx6* leads to failure of pancreatic islet development and diabetes in zebra fish and humans [21,22]. *Rfx7* deletion was recently shown to affect ciliogenesis in the developing neural tube in *Xenopus*, where it functions developmentally upstream of *Rfx4* [23]. *Rfx8* has only been identified by genomic similarity, and functional studies have not been reported (http://www.uniprot.org/uniprot/D3YU81).

In non-mammalian vertebrates *Rfx2* is crucial for the differentiation of cells carrying motile cilia and for the development of left/right asymmetry [24–27]. In contrast, the function for *Rfx2* in mammals is not well established. Initial expression profiles established in mice demonstrated extremely high transcript levels in testis compared to other organs [28]. This was amply confirmed by multiple genome wide studies (http://germonline.org/). Developmental studies revealed an initial increase in *Rfx2* mRNA during the meiotic phase of spermatogenesis [29–31]. This was confirmed at the protein level by immunohistology, showing that RFX2 is restricted to the germ line and not detected in Sertoli cells [32]. RFX2 has also been implicated in the upregulation of several genes in that period [33,34]. Regarding its own regulation, the *Rfx2* promoter region contains multiple binding sites for the MYB family of transcriptional regulators [33], of which *A-Myb* (*Mybl1*) is critically required for spermatocytes to complete meiosis [35,36]. Because RFX2 expression is greatly reduced in *A-Myb* deficient mice, it was predicted that *Rfx2* is controlled by A-MYB and in turn controls an unknown population of downstream genes important for spermatogenesis [37].

To test this last prediction we inactivated the *Rfx2* gene in mice. *Rfx2* ^*-/-*^ mice develop normally and are healthy, but males are sterile whereas fertility of female mice is not affected. We observed that spermatogenesis proceeded essentially normally through meiosis, but that all spermatids failed to progress past the round cell stage and were shed from the germinal epithelium at approximately step 7 of development. We observed a general defect in Golgi organization and acrosome formation associated with complete failure of axoneme elongation. RNA-seq based transcription-profiling performed with *Rfx2*-deficient testes at P21 and P30 identified over 100 and 600 genes, respectively, that were downregulated greater than two-fold. ChIP-Seq analysis at P21 identified many of these as being direct RFX2 targets, with a substantial number of them sharing a developmental expression pattern similar to that of *Rfx2*. Interestingly, this large group of downregulated genes shares almost no overlap with genes regulated by other transcription factors required for spermiogenesis [38–40]. These results clearly place RFX2 among the major transcriptional regulators of the haploid phase of sperm formation.

## Results

### Generation of *Rfx2* ^*-/-*^ mice

**Fig 1:**
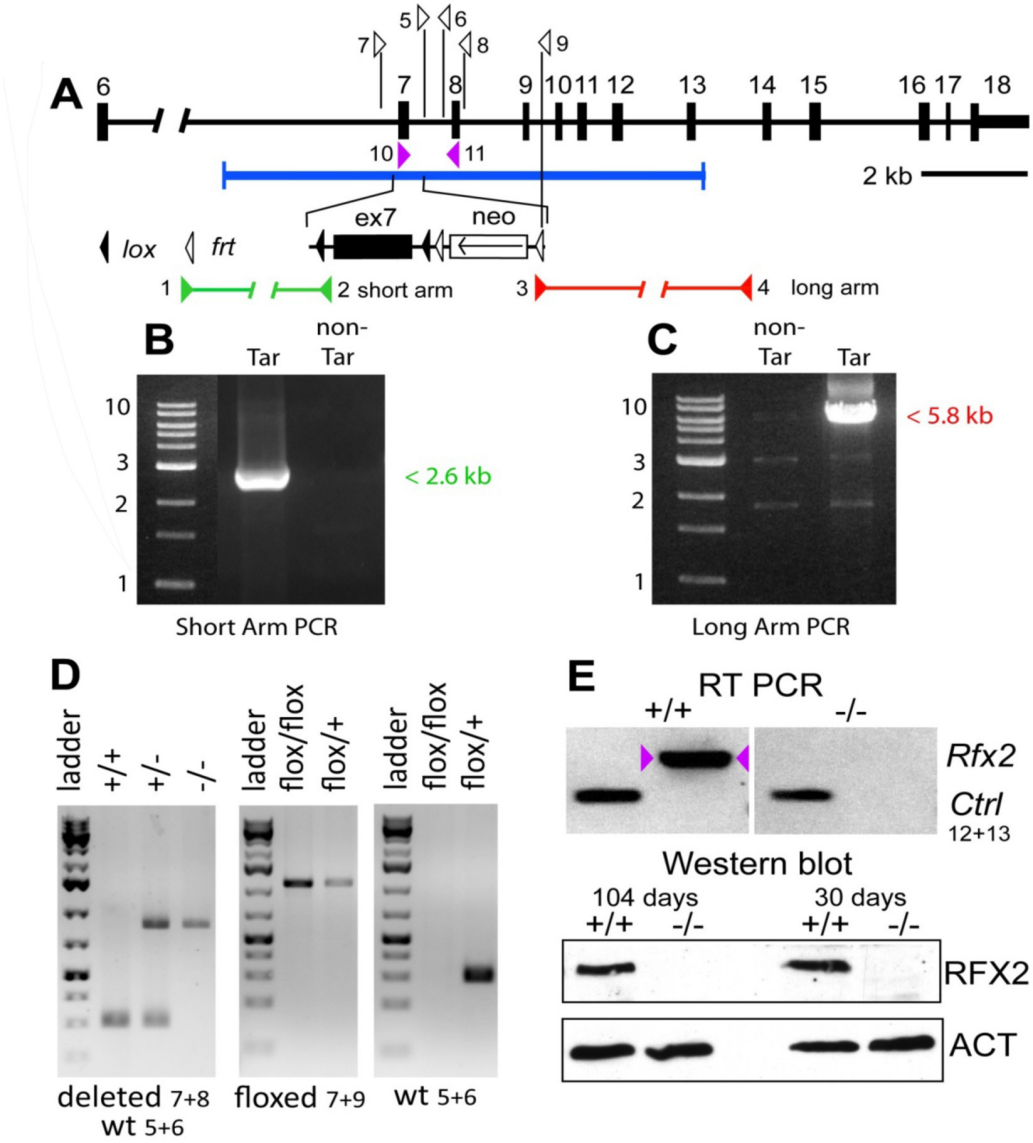
Generation *Rfx2* ^*-/-*^ *mice*. The mouse *Rfx2* gene is depicted approximately to scale from exon 6 onward (A). Below is shown the region of homology of the targeting vector, with the details of the floxed exon 7 enlarged. Locations of PCR primers are indicated above and their sequences are provided in S5 Table. ES cell clones were tested for targeted insertions using primer sets with one member lying outside the region of homology either up (B) or downstream (C) of *Rfx2* sequences present in the targeting vector. Mice were genotyped by tail biopsy using primer-pairs specific to the WT, floxed or deleted alleles (D). Reverse transcriptase PCR using testis cDNA, and a primer set anchored in exons 7 and 8, confirmed that exon 7 was absent in homozygous *Rfx2* ^-/-^ mice (E). Control amplification (Ctrl) detected a ubiquitously expressed component of the mitochondrial F1 ATP synthase complex (*Atp5a1*). Western blotting of testis protein extracts from mice of 30 or 104 days of age confirmed that full length RFX2 was not detected in homozygous *Rfx2* ^-/-^ mice (E). Actin (ACT) was used as control.

**Fig 2:**
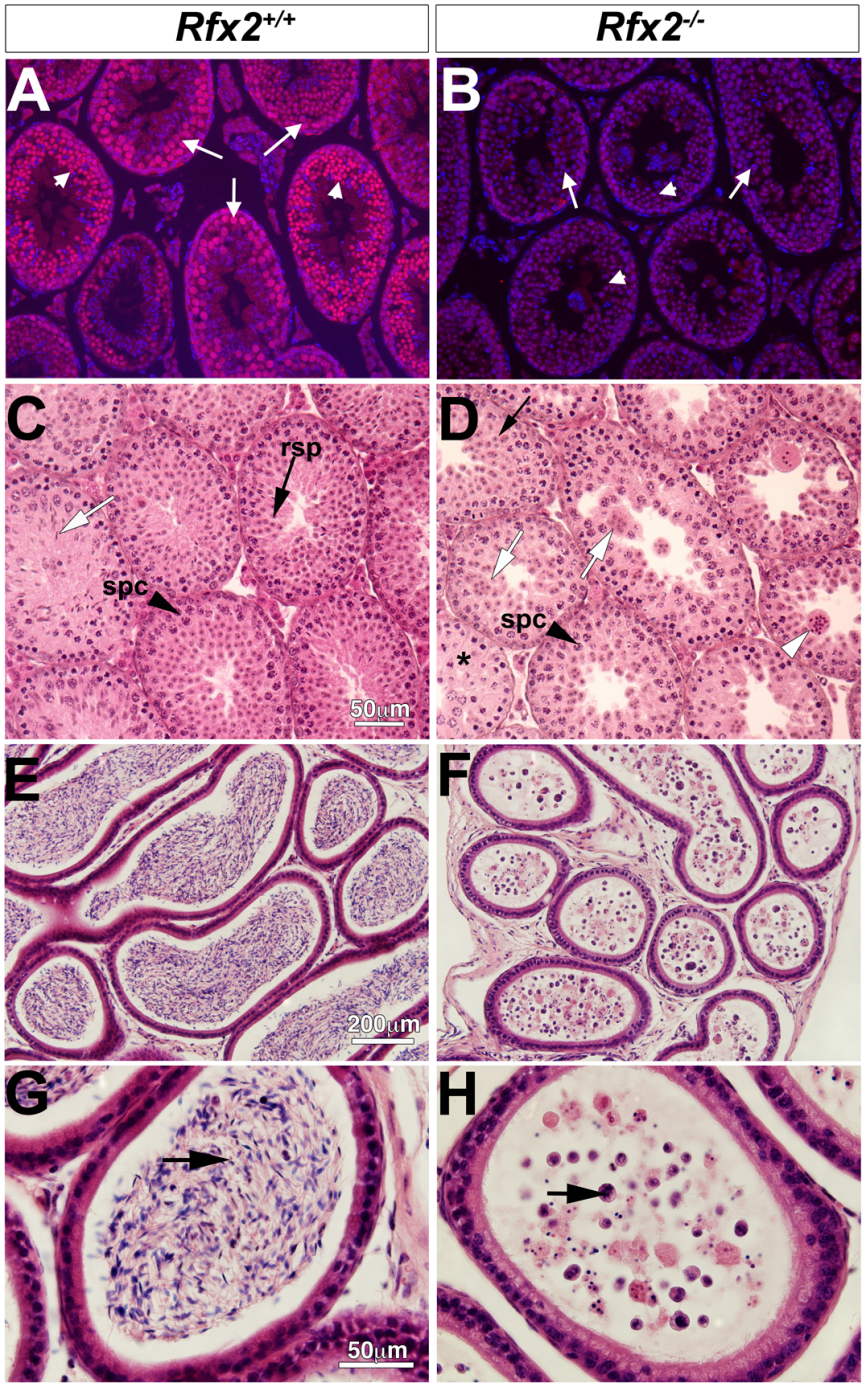
Spermatogenesis is arrested in *Rfx2* ^*-/-*^ mice. (A-B) Testis sections from *Rfx2* ^-/-^ and *Rfx2* ^+/+^ mice were stained with anti-RFX2 antibodies. RFX2 is expressed in pachytene spermatocytes (arrows) and early round spermatids (arrowheads). No RFX2 staining is observed in *Rfx2* ^*-/-*^ sections. (C-D) H&E stained sections from 30 day old *Rfx2* ^+/+^ and *Rfx2* ^-/-^ littermates are shown. (C) Seminiferous tubules from *Rfx2* ^+/+^ males show orderly development of germ cells of different stages including spermatocytes (black arrowhead, spc), round spermatids (black arrow, rsp), and late stage of spermatids with condensed nuclei (white arrow). (D) In striking contrast, *Rfx2* ^-/-^ mice exhibit an arrest in differentiation of haploid cells at approximately step 7 of the round spermatid phase (black arrow), without evidence of flagellum formation or condensation of the nucleus. Spermatids start to fuse at this stage (white arrow) leading to the formation of giant multinucleated cells that subsequently develop highly condensed nuclei (white arrowhead). Spermatocytes are normally present (spc, black arrowhead) and undergo meiosis. (E-G) In 3 month-old *Rfx2* ^*+/+*^ mice, the cauda region of the epididymis is filled with mature sperm (E, arrow in G). (F and H) The cauda epididymis of a 3-month-old *Rfx2* ^-/-^ mouse contains no mature sperm and only cell remnants (arrow).

A null allele was created for *Rfx2* by flanking exon 7 with loxP sites, creating targeted ES cells and then chimeric mice that were subsequently bred to obtain germline transmission. Subsequent Cre-induced recombination resulted in the excision of exon 7 (see Materials and Methods and Fig 1). Removal of exon 7 deletes most of the coding region for the DNA binding domain, and is predicted to change the reading frame and introduce premature out-of-frame stop codons if either exon 5 or 6 is spliced to exon 8, 9 or 10. Interbreeding of heterozygous exon 7 deleted mice generated the three expected genotypes for both sexes in the expected Mendelian ratios. Exon 7 was shown to be absent by RT-PCR of testis RNA from *Rfx2* ^*-/-*^ mice (Fig 1E). RNA-Seq experiments (see below) confirmed the deletion as no reads were obtained for exon 7. RFX2 protein was undetectable by Western blotting (Fig 1E) and immunohistology (Fig 2A and 2B).

### Spermatogenesis is arrested in *Rfx2* ^*-/-*^ mice

*Rfx2* ^*-/-*^ pups grow normally and show no developmental defects. Hence *Rfx2* ^*-/-*^ mice do not exhibit the ciliopathy hallmarks observed in *Rfx3* ^*-/-*^ mice or other phenotypes observed for *Rfx*-deficient mice [13,14,17–19,21]. *Rfx2* ^*-/-*^ males are sterile, but *Rfx2* ^*-/-*^ females have no obvious reproductive defects, as *Rfx2* ^*-/-*^ females (n = 7) produced litters that were comparable to *Rfx2* ^*+/-*^ females (average litter size was 8 for both genotypes). Initial morphological characterization showed that young adult *Rfx2* ^*-/-*^ males had slightly smaller testes but that the remainder of the male reproductive tract and accessory glands, such as seminal vesicles, appeared normal. Microscopic examination (Fig 2E–2H) revealed that the epididymis contained no sperm but instead large numbers of degenerating small cells and cell debris. The overall microscopic architecture of the testis was normal, but there was a complete block of spermatogenesis prior to the point where round spermatids should begin to elongate and develop characteristic features of spermatozoa (Fig 2C and 2D). Cells at this stage instead formed multinucleated cells with condensed nuclei, also called symplasts in some studies [41], and were released from the tubules to flow into the epididymis (white arrowhead, Fig 2B).

**Fig 3:**
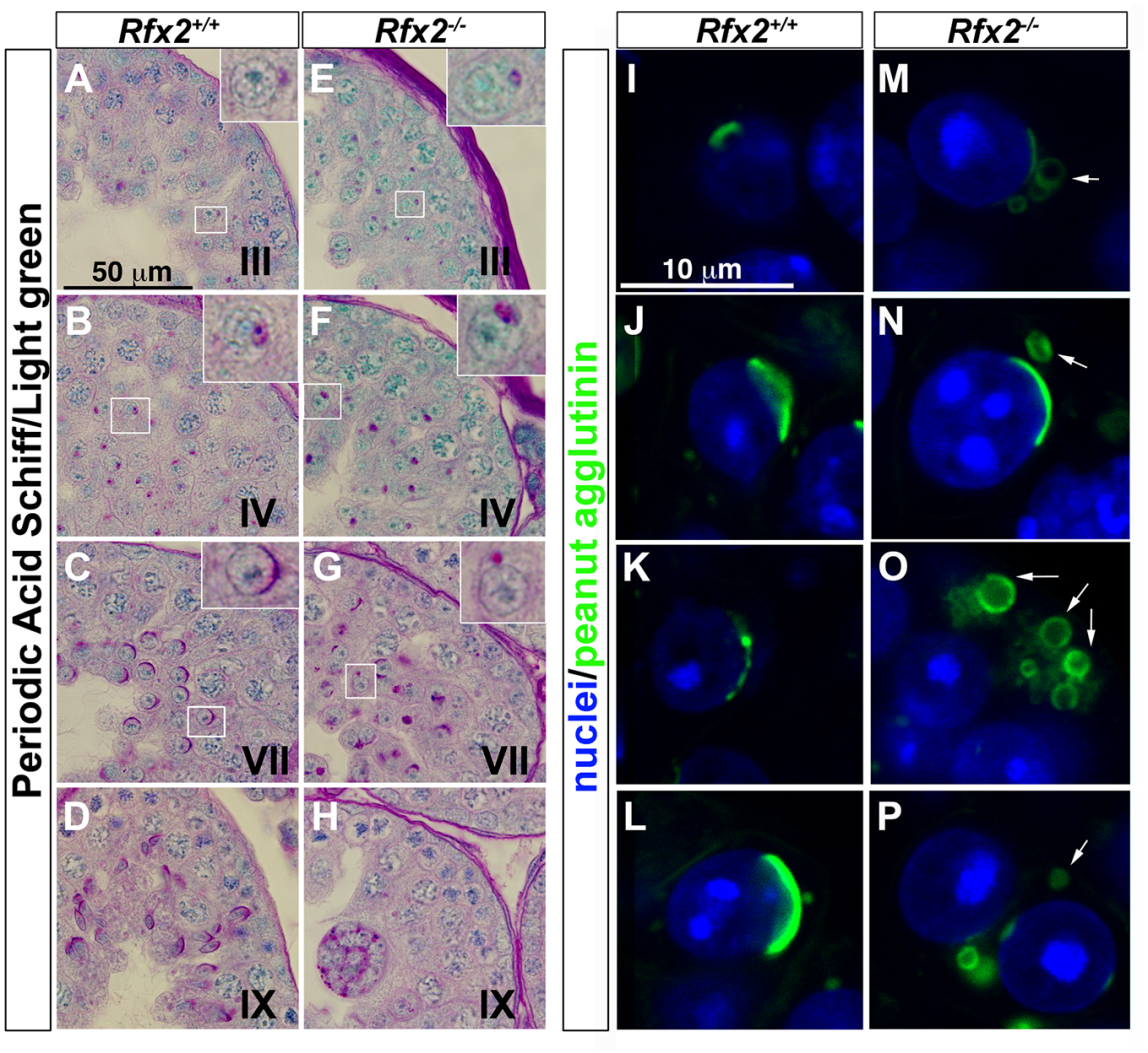
The characteristic acrosome organelle fails to develop properly in *Rfx2* ^-/-^ mice. (A-H) Periodic acid shiff (PAS) / light green stained segments of testis sections from *Rfx2* ^*+/+*^ mice (A-D) and comparable sections from *Rfx2* ^*-/-*^ mice (E-H). Developmental stages of the seminiferous tubules are noted on each panel. Insets show a 3-fold magnification of a spermatid from the respective section (box). Insets show in: (A, E) step 2 spermatids, (B, F) step 5 spermatids, (C, G) step 7 spermatids. (I-P) High power views of individual cells with the acrosomes stained by fluorescently-tagged peanut agglutinin. For *Rfx2* ^*+/+*^ sections, different step of acrosome development are shown (I: step 2, J-L: step 6–7 spermatids). For *Rfx2* ^*-/-*^ sections, variations in acrosome morphology are shown for spermatids that are about to arrest (M-P). In the majority of cases the acrosome fails to spread normally over the anterior end of the nucleus and frequently occurs as unattached vesicles (arrows).

One of the most readily observed changes between the end of meiosis and the beginning of spermatid elongation is formation of the acrosome, which is characteristically stained by the periodic acid-Schiff (PAS) reagent due to its high glycoprotein content. Light microscopic examination of testes from 30-day-old *Rfx2* ^*-/-*^ mice, in which the first wave of developing germ cells has only recently encountered the point of arrest, showed that Golgi-derived proacrosomic vesicles appear as expected. However, they frequently fail to attach to the nucleus, and the acrosome becomes increasingly disorganized in appearance (Fig 3A–3H). This can be seen in greater detail using fluorescently tagged peanut agglutinin to label acrosomes (Fig 3I–3P). Almost no spermatids showing a typical cup-shaped acrosome spread over the nucleus, as observed in wild type (WT) step 7 spermatids, can be visualized in *Rfx2* ^*-/-*^ testis sections. At approximately step 6–7 the mutant spermatids begin to fuse into multinucleate giant cells.

### Arrested spermatid differentiation is not associated with increased apoptosis or accelerated accumulation of transition proteins in the nuclei

WT and *Rfx2* ^-/-^ testis sections were stained by the TUNEL technique to ascertain if the arrest in spermatid development was associated with increased apoptosis. No difference in the distribution or number of TUNEL positive cells was detected (S1A and S1B Fig). Differences in the patterns of phospho-H2AX staining in round spermatids or the condensed nuclei in multi-nucleated bodies were also not observed (S1C Fig). Nuclear condensation in multinucleated cells is thus not due to induction of apoptosis.

We next examined whether the appearance of smaller nuclei among the multi-nucleate bodies could be due to premature or aberrant synthesis of proteins involved in the normal histone to protamine transition. This stepwise process involves multiple proteins including transition proteins TNP1 and TNP2, major players in this process [42]. mRNAs for TNP1 and 2 are not reduced in the knockout mice, as revealed by our RNA-Seq analysis described below. Like many transcripts present at the end of the round spermatid stage, these mRNAs are translationally repressed until the appropriate time. However, TNP1 was not detected by Western blotting at all developmental stages (S2A Fig), whereas TNP2 was present in *Rfx2* ^*-/-*^ testis even though spermatid development does not reach the stage (steps 9–10) at which it first appears in WT animals (S2A Fig). When sections were stained for TNP2 it was detected largely over the cytoplasm of terminal stage *Rfx2* ^*-/-*^ spermatids, in contrast with its nuclear localization in *Rfx2* ^*+/+*^ mice (S2B and S2C Fig). This suggests that disorganized development may proceed to the point where translational repression of TNP2 mRNA is released, allowing accumulation of TNP2 protein, but that the latter fails to be transported efficiently into the nucleus. These results suggest that premature nuclear accumulation of transition proteins is not likely to be responsible for nuclear condensation in multinucleated cells. We cannot exclude that altered protamine levels could be involved in nuclei condensation, but this would likely be an indirect consequences of earlier nuclear defects as protamines are produced after the spermatid arrest observed in *Rfx2-/-* testes.

### Perturbed proacrosomal vesicle and axoneme formation in *Rfx2* ^*-/-*^ spermatids

**Fig 4:**
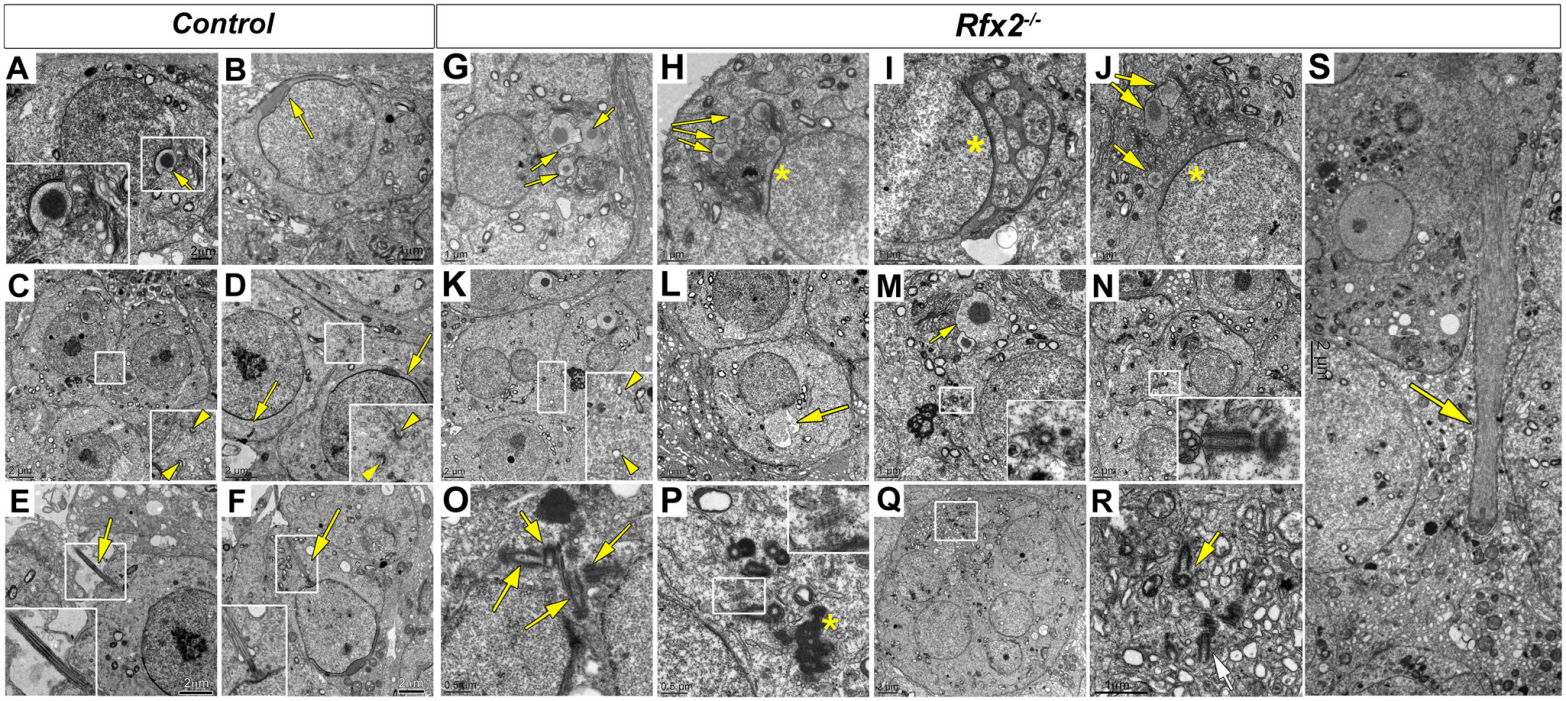
Ultrastructural defects and perturbed Golgi organization in *Rfx2* ^*-/-*^ testes. Transmission electron microscopy images of control (A-F) or *Rfx2* ^*-/-*^ (G-S) spermatids. (A) The acrosome vesicle (arrow, 2-fold magnified inset) has fused with the nucleus and is beginning to flatten at step 4. (B) As development progresses toward the end of the cap phase, acrosomes have flattened and spread over approximately half of the nuclear surface in step 7 spermatids (arrow). (C-D) cytoplasmic bridges (between two arrowheads in insets showing 2-fold enlargements) are visible between two spermatids. The acrosomal cap is marked with arrows. (E) Developing axonemes (arrows, insets show two-fold enlargements of the axonemes) are prevalent among late stage round spermatids and can be seen clearly on an early elongating spermatid of step 9 (F). (G-S) For *Rfx2* ^*-/-*^ sections various aberrant morphologies are shown for cells at about the point of developmental arrest. (G-J) Examples of aberrant acrosomes observed in *Rfx2* ^*-/-*^ spermatids. (G) Multiple acrosomic vesicles are present without attachment to the nucleus (arrows) or (H) coexisting with some degree of an attached and flattened acrosome (asterisk). (I) An atypical acrosome (asterisk) has attached and spread but contains multiple cytoplasmic inclusions. (J) Another example of multiple unattached vesicles (arrows). (K) Cytoplasmic bridges (between two arrowheads, 2-fold magnification inset) are apparently normal until spermatids fuse, forming round huge multinucleated cells, and cytoplasmic bridges disappear. (L) Nucleus with a protruding aneurysm-like rupture (arrow). (M, N) Cells contain isolated centriole pairs that have not generated axonemes (inserts). In (M), arrow points to the acrosomal vesicle. (O, P, Q, R) Within multinucleated giant cells, clusters of centrioles occur (arrows), but without associated axonemes. (Q, R) A giant multinucleated cell contains a cluster of centrioles (box enlarged in R, arrows). Ciliary rootlets are visible (2-fold enlarged inset in P), the asterisk indicates the chromatoid body. (S) A large bundle of microtubules that could be an ectopic manchette (arrow). Sizes of scale bars are indicated in individual panels.

EM analysis was performed to characterize in more detail the cellular defects observed in *Rfx2* ^*-/-*^ spermatids. Shortly after meiosis in WT spermatids, cell polarity is established such that the Golgi apparatus is oriented with the trans-Golgi facing the nucleus. This favors the transport of budding vesicles to the nuclear surface, where they attach to the nuclear lamina [43,44]. The migration of Golgi vesicles to the nucleus is guided by microtubule tracks and mediated by molecular motors and RAB adapter proteins [45–48]. At the opposite pole of the cell, a single axoneme develops near the plasma membrane from one of the two centrioles and gradually elongates as the basal body migrates inwards toward the nucleus [43]. These processes do not occur correctly in *Rfx2* ^*-/-*^ mice. The Golgi frequently appears disoriented such that dense-cored proacrosomic vesicles migrate away from the nucleus (Fig 4G–4M). These vesicles fuse to form large dense-cored discs that do not attach to the nucleus (Fig 4G, 4H, 4I and 4J). In other cases, vesicles have fused with the nucleus to form a partial acrosomal cap that only rarely spreads to cover half of the anterior end of the nucleus, as is the case for WT mice (compare Fig 4B–4D with 4H–4J). Occasionally, highly atypical structures are found, such as the development of an acrosome having apparently engulfed multiple vesicles containing cytoplasm (Fig 4I). Nuclei more often show protruding aneurysm-like ruptures in *Rfx2* ^*-/-*^ cells (Fig 4L). No signs of developing axonemes are found, although these are evident in WT mice (compare Fig 4E and 4F with 4M and 4N and insets). This is in agreement with the absence of acetylated tubulin stained flagella in the lumen of seminiferous tubules (S3 Fig). Interestingly, centriole pairs are found, and can occur in clusters in the giant multinucleated cells (Fig 4N–4R).

In WT spermatids, once the acrosome cap has covered slightly more than half the nucleus, the nucleus and associated acrosome move to contact the plasma membrane, which becomes the site of new specialized junctions, the apical ectoplasmic specialization, between the germ cells and Sertoli cells [49,50]. In WT mice, these will control Sertoli cell—germ cell connections until the normal release of mature spermatids at spermiation. In *Rfx2* ^*-/-*^ spermatids the nucleus/acrosome is not observed to merge with the plasma membrane, suggesting that these junctions do not form. Coincident with this failure, mutant spermatids undergo fusion to form large multinucleate cells. Prior to this point, the mutant spermatids, like all differentiating male germ cells, maintain open cytoplasmic connections via so-called cytoplasmic bridges characterized by well-defined boundaries [51] (Fig 4K). Breakdown of the supporting boundaries of these bridges presumably accompanies the formation of giant cells, but this could not be formally demonstrated from the ultrastructure images, suggesting that once begun this is a rapid process. With the failure to form and maintain the ectoplasmic specialization junctions with Sertoli cells, the spermatids, now largely or entirely in the form of giant cells, are released to pass out of the testis and into the epididymis.

Another prominent ultrastructural feature of the maturing round spermatid is formation of the manchette, a dense arrangement of microtubules attached to the outer edge of the nucleus in a ring just below the border of the acrosome. The manchette is believed to be important for the nuclear shaping that marks the end of the round phase of spermatid differentiation [52,53]. Although no manchette is observed in *Rfx2* ^*-/-*^ spermatids, dense microtubule clusters are seen in the multinucleated cells, perhaps representing ectopic manchette formation (Fig 4S).

**Fig 5:**
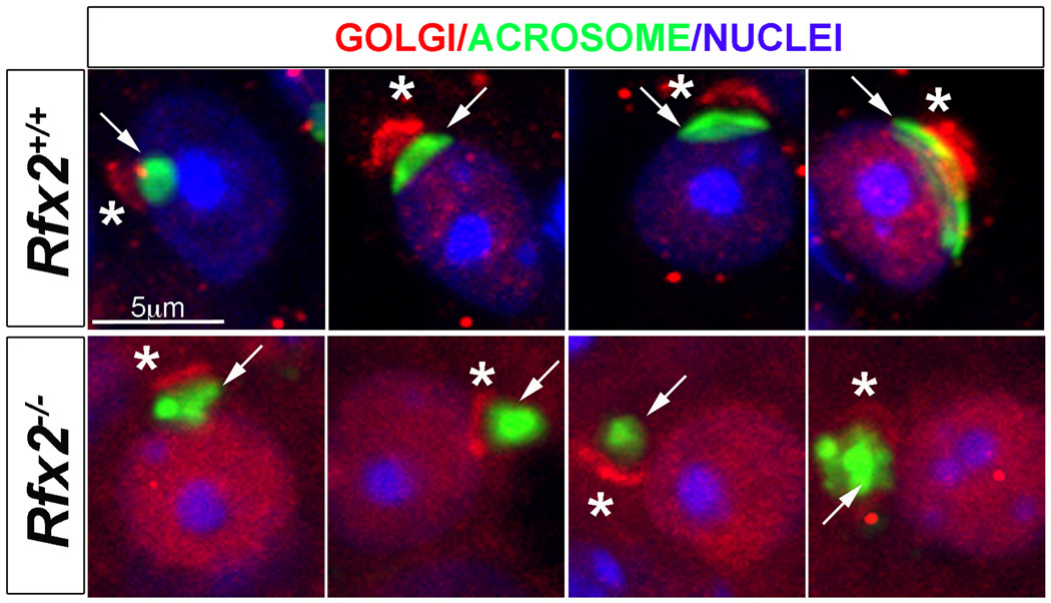
Testis sections from P30 *Rfx2* ^+/+^ or *Rfx2* ^*-/-*^ mice stained for the cis-Golgi compartment (GM130 antibody, red), the acrosome (peanut agglutinin, green) or nuclei (Dapi, blue). In WT spermatids, the Golgi compartment (asterisks) is concentrated at one pole of the nuclei above the forming acrosome (arrow) on the nucleus. In *Rfx2* ^*-/-*^ testes, GM130 staining (asterisks) is observed adjacent to the nuclei and acrosome (arrow) and can be correctly orientated in a few situations (left panel), but the overall orientation of the Golgi and acrosome is most frequently disturbed with the cis-Golgi being (from left to right) between the acrosome and the nucleus, apposed to the Golgi and the nucleus, or surrounding the acrosome.

Staining of testis sections for the cis-Golgi marker GM130 revealed a severely altered GM130 distribution in *Rfx2* ^*-/-*^ mice (Fig 5). Whereas GM130 is mainly concentrated at one pole of WT spermatids, it is also diffusely distributed around nuclei in *Rfx2* ^*-/-*^ spermatids, illustrating an altered Golgi re-distribution at the onset of the haploid stage.

### A wide range of mRNAs is mis-regulated in *Rfx2* ^*-/-*^ spermatids

**Fig 6:**
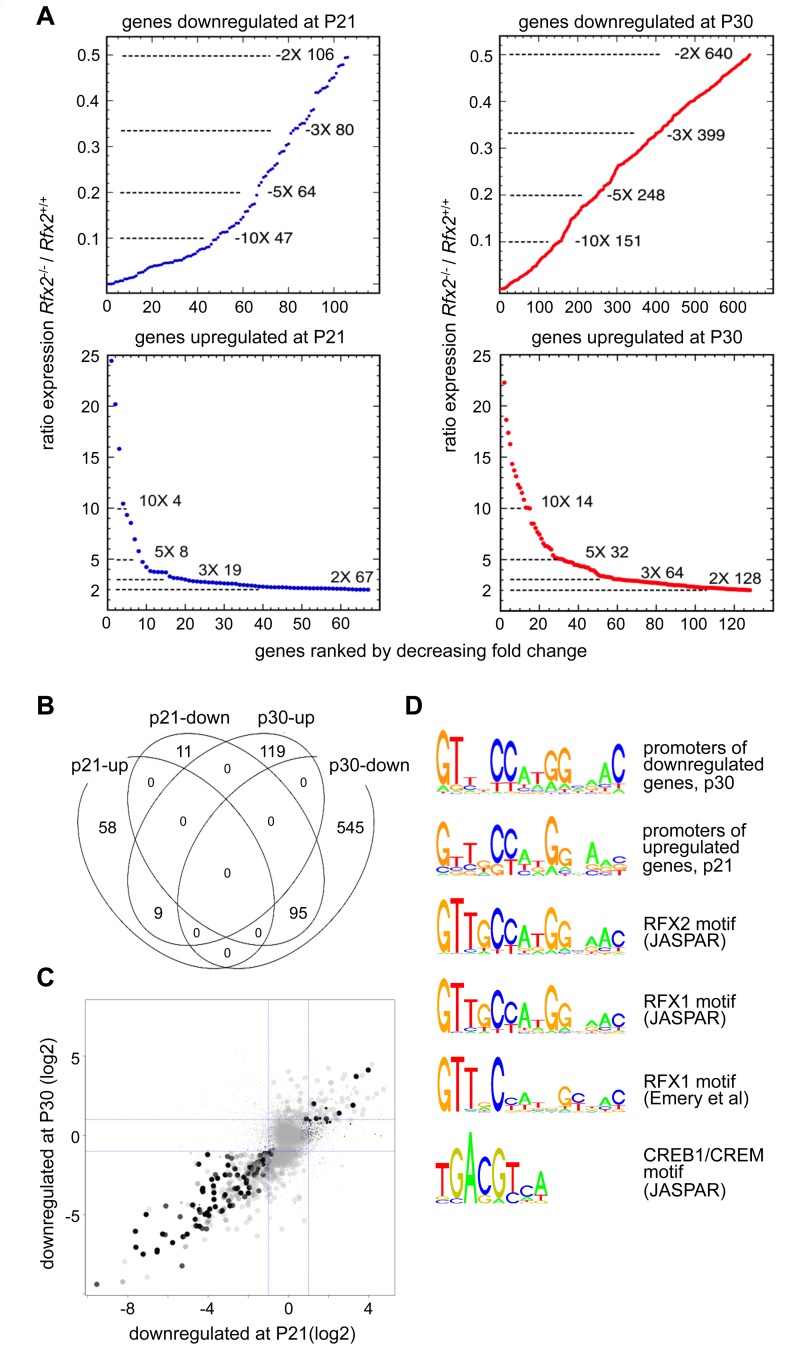
Perturbed gene expression in *Rfx2* ^-/-^ testis. (A) Plots of the cumulative number of downregulated genes as a function of their expression level in *Rfx2* ^-/-^ relative to *Rfx2* ^+/+^ testis: (top) downregulated genes at P21 and P30; (bottom) genes upregulated at P21 and P30. (B) Venn diagram representing the overlap between each set of differentially expressed genes. (C) Differentially expressed genes were plotted according to their fold-change (log2) at P21 and P30. Dot size is inversely proportional to the p-value at P21. Black to grey scale is inversely proportional to the p-value at P30. (D) Motifs that are significantly enriched in the promoters of P30 downregulated or P21 upregulated genes are compared with the X motifs defined for RFX1 and RFX2 in JASPAR, and the X motif identified experimentally for RFX1 [8]. The motif identified in JASPAR for CREB1/CREM is also reported, to show the absence of homology with RFX binding motifs.

RNA-Seq based transcriptome profiling of testis cells from *Rfx2* ^*+/+*^ and *Rfx2* ^*-/-*^ mice was performed to assess the consequences of the loss of RFX2 function. Animals were chosen at postnatal days 21 (P21) and 29–30, henceforth referred to as (P30) as there are no significant changes in germ cell populations in this time window. At P21, the most advanced tubules contain only very early round spermatids while pachytene spermatocytes are plentiful. At this stage, defects in spermatogenesis are not yet histologically evident in *Rfx2* ^-/-^ mice. At P30, the first wave of germ cells has largely reached the point of arrest observed in *Rfx2* ^-/-^ mice, but potential long term cumulative effects on seminiferous tubules should be minimal. Global representations of RNA-Seq experiments comparing the transcriptomes of WT and *Rfx2* ^-/-^ testes at days P21 and P30 are shown in S4 Fig. At P21, 106 genes were down regulated (p < 0.01) greater than 2-fold, of which 47 were decreased more than 10-fold (Fig 6A, S1 Table). By P30, the number of 2-fold downregulated genes increased to 640, with 151 being decreased 10-fold or more (Fig 6A, S1 Table). Most genes downregulated at P21 were also downregulated at P30: 95 genes were affected at both time points (Fig 6B and 6C). Markedly fewer genes were upregulated more than 2-fold: 67 genes at P21 and 128 genes at P30 (Fig 6A, S1 Table). Only 9 genes were upregulated significantly at both time points (Fig 6A, S1 Table).

**Fig 7:**
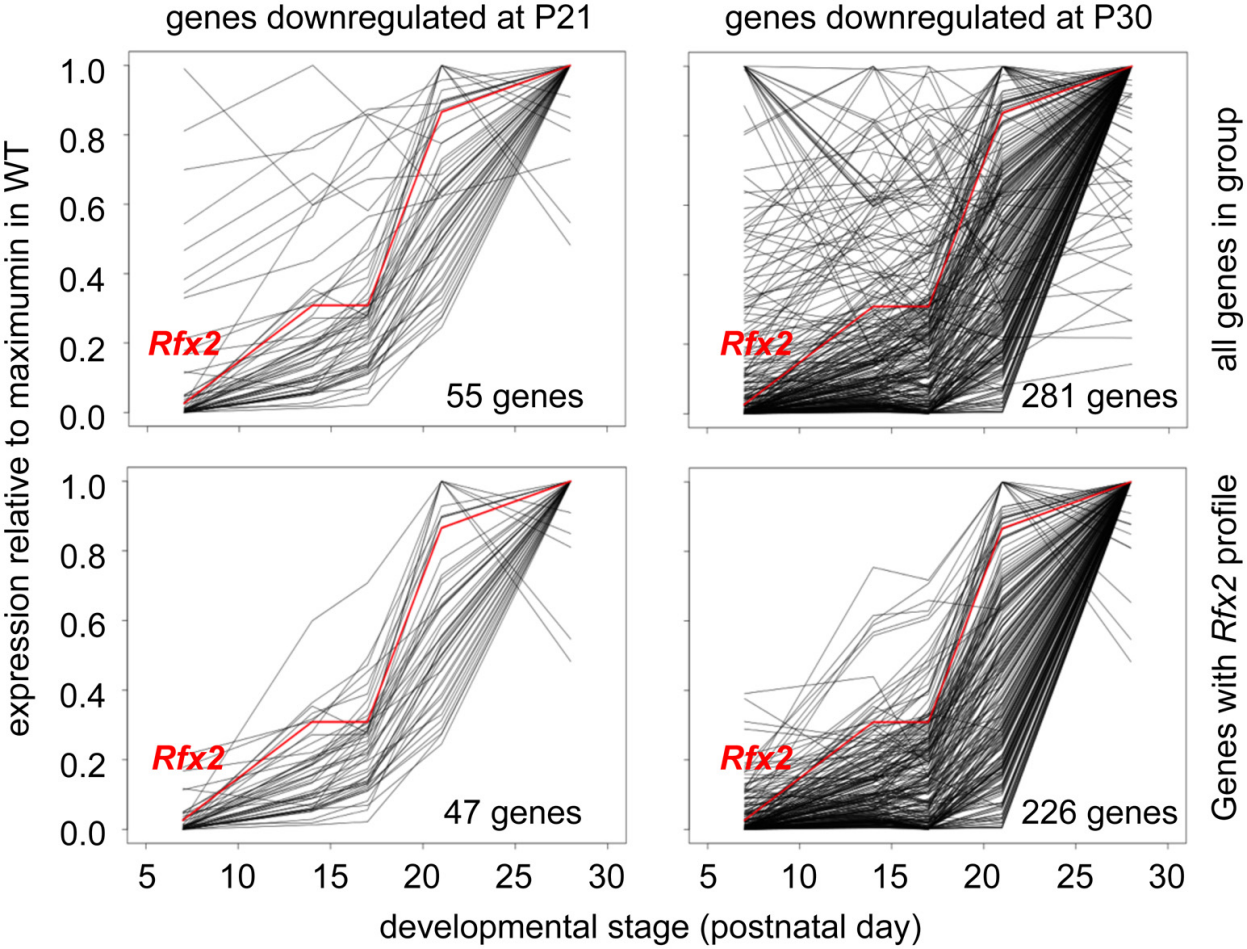
Developmental expression profiles of genes downregulated in *Rfx2* ^-/-^ testis. Expression patterns derived from [29], are shown for genes that are downregulated in *Rfx2* ^-/-^ testis at P21 (left plots) or P30 (right plots). The plots show the expression patterns for all genes in the group (top) or for genes exhibiting expression patterns that are the most consistent with activation by RFX2 (lower). Gene totals are less than in Fig 6 because genes exhibiting unreliably low read values in the Laiho et al. data set were not included.

To identify genes that are downregulated in *Rfx2* ^-/-^ testis and exhibit a developmental expression program consistent with activation by RFX2 in WT testis, we used the WT expression data described in a recent RNA-Seq analysis of mouse spermatogenesis [29]. *Rfx2* expression first increases significantly by day 14, as cells reach early-mid pachytene, and then increases dramatically between days 17 and 21, as the leading cells complete meiosis (Fig 7). Among genes that are downregulated in *Rfx2* ^-/-^ testes and exhibit statistically reliable expression profiles in the data of Laiho et al [29], the majority (47/55 at P21 and 226/281 at P31) exhibits expression profiles during spermatogenesis consistent with activation by *Rfx2*, (Fig 7, S2 Table).

Searches for transcription factor binding sites in the promoters of differentially expressed genes, revealed that RFX binding motifs are highly enriched in genes that are upregulated at P21 or downregulated at P21 and/or P30 (S5 Fig). Similarly, an unbiased motif discovery approach identified a 14 bp inverted repeat exhibiting strong homology to the consensus RFX binding site (X box) in the promoter regions (-500 to + 50 bp relative to the predicted transcription start site, TSS) of genes that are upregulated at P21 and downregulated at P30 in *Rfx2* ^-/-^ testis (Fig 6D). Overall, 159 differentially expressed genes contain an X-box in their promoter regions (S1 Table). These observations suggest that RFX2 directly regulates these genes.

### Identification of target genes regulated directly by RFX2

**Fig 8:**
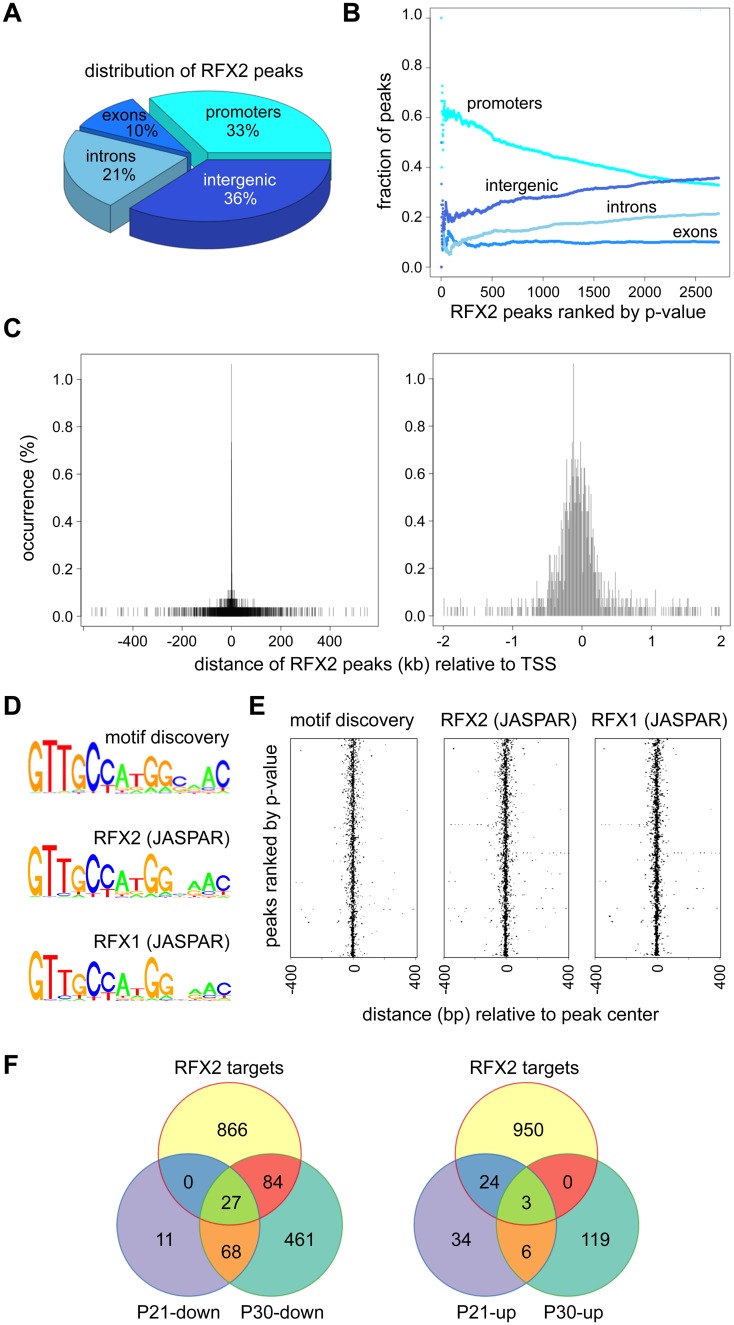
ChIP-seq analysis of RFX2 target genes in mouse testis. (A) Distribution of RFX2 binding peaks in different genomic regions. (B) Fractions of peaks mapping in the different regions relative to the p-value of the peaks. Peaks are ranked according to increasing p-value. (C) Distribution of peak centers relative to the TSS for genes having peaks within promoter regions. Peaks are mostly centered near the TSS. (D) A motif that is significantly enriched in the promoters of Rfx2 downregulated target genes is compared with the X motifs defined for RFX1 and RFX2 in JASPAR. (E) Position of the *de novo* identified motif (motif discovery) and predicted RFX1 or RFX2 motifs relative to the TSS of RFX2 target genes. All three motifs cluster near the TSS. (F) Venn diagram showing the overlap between RFX2 targets (identified by ChIP-Seq) and differentially expressed genes at P21 and P30.

To identify genes that are regulated directly by RFX2 in spermatocytes and early spermatids, we carried out chromatin immunoprecipitation followed by high throughput sequencing (ChIP-Seq), using dissociated germ cell preparations from P21 WT mice. We identified nearly 3,000 reproducible and statistically-significant peaks corresponding to binding of RFX2 (Fig 8). Of these peaks, about 1/3 (977) were located within presumptive promoter regions (-500 to- +50), whereas the remaining were distributed in introns, exons and intergenic regions (Fig 8A). The most robust (lowest p-value) peaks were preferentially enriched in promoter regions (Fig 8B). Representative promoter peaks are shown in S6 Fig. The distribution of promoter peaks showed a marked concentration near the TSS (Fig 8C). Genes with promoter peaks will henceforth be designated RFX2 targets.

An unbiased search for overrepresented sequence motifs in the ChIP peaks again identified a 14 nucleotide motif exhibiting an almost perfect match to consensus binding sites for RFX1 and RFX2 (Fig 8D), illustrating that the RFX2 binding motif found in spermatogenesis genes is not different from previously established X-box motifs. As expected, the RFX motifs are mostly located near the center of the ChIP peaks (Fig 8E). In summary, these data clearly show that RFX2 binding sites are located very close to the TSS in the majority of target genes.

**Fig 9:**
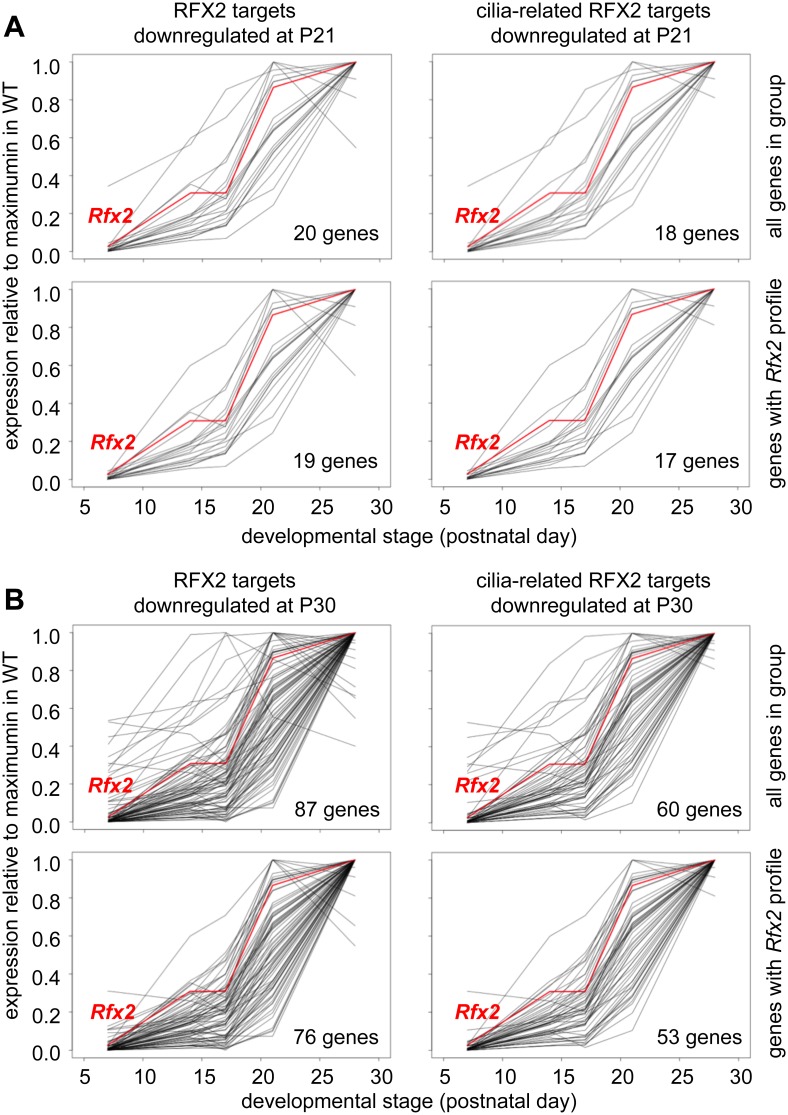
Developmental expression profiles of RFX2 target genes. Expression patterns derived from [29], are shown for target genes that are downregulated in *Rfx2* ^-/-^ testis at P21 (A) or P30 (B). The plots show the expression patterns for all genes in the group (top) or for genes exhibiting expression patterns that are the most consistent with activation by RFX2 (lower). Left panels show all genes in the group and right panels only cilia-related genes from each set.

Among RFX2 target genes identified by ChIP-seq, 138 were differentially expressed in *Rfx2* ^*-/-*^ testes (Fig 8F and S1 Table), 111 being downregulated and 27 being upregulated. This pinpoints these genes as primary RFX2 target genes in the mouse testis. A substantial fraction of these target genes exhibit expression patterns during spermatogenesis [29] consistent with activation by RFX2 (compare upper and lower left-hand panels in Fig 9A and 9B). These genes are therefore likely to be directly regulated by RFX2 in the testis.

### RFX2 target genes are involved in cilia assembly and function

**Fig 10:**
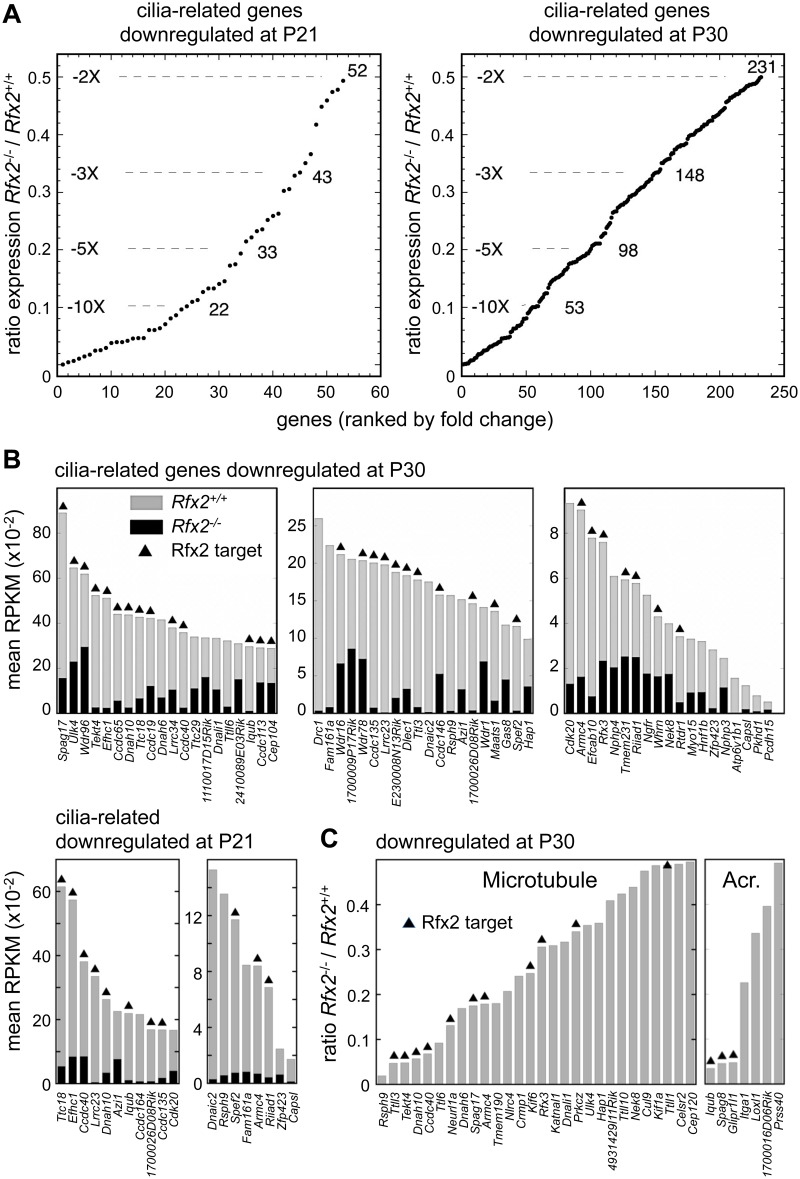
Downregulated genes having known or suspected functions related to cilia/flagellum formation, microtubule function or the acrosome. (A) Plot of the cumulative number of cilia-related genes as a function of their expression level in *Rfx2* ^-/-^ relative to *Rfx2* ^+/+^ testis. Downregulated genes analyzed are included in the Syscilia gold standard list [55] or have at least 1 high-confidence citation in the CilDB database. (B) Bars show the expression levels (RPKM, reads per Kb per million) in *Rfx2* ^*+/+*^ (grey) and *Rfx2* ^*-/-*^ (black) testis for downregulated genes included in the Syscilia Gold or Potential lists. RFX2 targets are indicated above the bars. Genes are ordered according to their expression level in WT mice. (C) Downregulated genes assigned to GO terms related to microtubule or the acrosome (GO function or compartment). Expression in *Rfx2* ^-/-^ testis is expressed relative to *Rfx2* ^+/+^ testis. RFX2 targets are indicated above the bars.

Gene ontology analysis (see Supplemental methods) revealed a specific and significant enrichment (p <0.001) in genes associated with GO terms related to ciliogenesis—such as cilium morphogenesis (GO:0060271), cilium organisation (GO:0044782), cilium assembly (GO:0042384) or cell projection GO:0042995—for both the set of direct RFX2 targets identified by ChIP-seq and the set of differentially expressed genes in *Rfx2* ^*-/-*^ testis (S7 and S8 Figs, S3 Table). We therefore examined the list of downregulated genes for those related to cilia using two reference lists. The first is the database “CilDB” [54]. The second is the “gold standard” list established by the Syscilia consortium [55]. CilDB provides a score corresponding to the number of times a gene is found with low, medium or high confidence in various studies aiming at identifying cilia-associated genes. Among genes that are downregulated in *Rfx2* ^*-/-*^ testis, 52/106 (49%) at P21 and 231/640 (36%) at P30 are potentially involved in ciliogenesis, as they are found with high confidence in at least one cilia-related study documented in the CilDB database and/or in the Syscilia gold standard list (Fig 10A, S1 Table). Of these, 43/52 at P21 (82%) and 148/231 at P30 (64%) are down regulated more than 3-fold (Fig 10A, S1 Table). Many of these downregulated cilia-related genes are direct targets of RFX2 (Fig 10B, S1 Table). Among genes that are both differentially expressed in *Rfx2* ^-/-^ testis and direct targets of RFX2, 80/138 (57%) are found with high confidence in at least one cilia-related study documented in the CilDB database and/or in the Syscilia gold standard list (S1 Table). Most of these genes exhibit developmental expression profiles [29], similar to *Rfx2* (Fig 9A and 9B right-hand panels). Examination of our ChIP-Seq data for a selection of cilia-associated target genes downregulated at P21 and P30 clearly showed peaks in their promoter regions (see S6 Fig for examples). Finally, among 28 cilia-related genes that are upregulated at P21, 11 are direct targets of RFX2 (S9A Fig). These results clearly indicate that genes involved in ciliogenesis are primary targets of RFX2 in the mouse testis.

### RFX2 regulates genes implicated in cell adhesion and intracellular trafficking

To understand how RFX2 deficiency could lead to specific Golgi-associated and acrosome-formation defects, we examined all GO terms associated with differentially expressed genes at P21 or P30. Many genes deregulated in *Rfx2* ^-/-^ testis were found to match to GO terms associated with intracellular transport, microtubule associated processes or small GTPase regulation, even though these terms were not significantly enriched. For example, of genes that are downregulated in *Rfx2* ^-/-^ testis, 35 belong to gene sets corresponding to the GO terms “microtubule” or “acrosome”, and a substantial fraction of these genes are direct RFX2 targets (Fig 10C). Impaired expression of these genes could contribute to defective cell polarization, proacrosomic vesicle migration and acrosome formation during the first steps of spermatid differentiation.

GO terms that are enriched with a p-value below threshold (0.01<p<0.001), include terms linked to cell-adhesion or cytoskeleton organization. Genes associated with these terms could account for some of the spermatid defects observed in *Rfx2* ^*-/-*^ mice, in which spermatids lose their contacts with adjacent Sertoli cells and form giant multinucleated cells. These genes include *Fndc3c1*, which is a paralogue of *Fndc3a*. The latter is mutated in the mouse *sys* strain (S4 Table), which has a phenotype strikingly similar to that of *Rfx2* ^-/-^ mice in that it is characterized by the formation of giant multinucleated cells referred to as symplasts [56]. Another interesting candidate is *Fascin* 1, which is down regulated both at P21 and P30, and is known to be involved in the ectoplasmic specialization required for spermatid-Sertoli cell contacts in the rat testis [57].

## Discussion

We demonstrate here that a targeted mutation of the gene encoding RFX2, one of the earliest identified members of the RFX family of transcription factors, leads to a complete block in mouse spermiogenesis. While over 400 mutations have been reported to affect spermatogenesis in the mouse [1], relatively few result in complete arrest of development at the end of the round spermatid stage [58] (S4 Table). Most of these mutations lead to defects in RNA processing or formation of RNA-protein complexes. A number also affect specific cell organelles or cell junctions. Prior to this report, only three of these mutations involved genes encoding TFs. Of these, two (*Tbpl1* and *Taf7l*) [40,59] encode variants of general transcription factors (GTFs), leaving CREM as the only sequence/gene-specific TF established to have a pivotal role in controlling spermiogenesis at the round spermatid stage [60,61]. Our results thus identify RFX2 as a second essential master TF implicated in this stage of spermatogenesis. Of note, none of the transcriptional regulators known to be required for spermiogenesis (see S4 Table) are affected by *Rfx2* disruption.

### RFX2, CREM and TAF7l control largely non-overlapping gene sets

Genome wide analyses of their effects on transcriptional programs in testis have been performed for mutations in *Crem* and *Taf7l* [39,40]. *Crem* occupies a special place among genes encoding transcriptional regulators of spermatogenesis. In the testis it produces a specific splice variant encoding CREM-tau, a unique isoform of the cAMP-responsive element modulator (CREM), which comprises a family of isoforms that bind to the cAMP response element (CRE). While most of the splice variants produce inhibitory factors, CREM-tau is stimulatory and appears late in meiosis [62]. *Crem* was one of the earliest targets of engineered mutations [60,61] and proved to be essential for expression of a large number of classic genes expressed in the haploid phase of spermatogenesis. A recent genome wide study identified 627 genes that were downregulated greater than 2-fold in *Crem* ^*-/-*^ testis [39]. Of these CREM-regulated genes, 277 were shown by ChIP-Seq experiments to be direct targets of CREM in male germ cells [63]. Only 34 of the CREM-regulated genes and 9 of the CREM-occupied genes are downregulated greater than 2-fold in *Rfx2*-deficient testis (S10A Fig). Furthermore, RFX2 and CREM regulated gene sets exhibit largely distinct patterns of expression during spermatogenesis (S10B Fig). Direct RFX2 target genes tend to parallel the expression pattern of RFX2 itself, starting from little or no significant expression at P7 and rising to half maximal by or before P21. In contrast, direct CREM targets have extremely variable expression at P7 but tend to share large increases after P21 (S10B Fig). Genes that depend directly on RFX2 or CREM thus constitute nearly distinct sets.

Spermatogenesis exhibits a striking dependence on variants of the Pol II GTFs [64], which include TBL1 (TRF2) [59], TAF4b [65], TAF7l [40], and GTF2a1l (ALF) [66]. TAF7l, a variant of TAF7, is a component of TFIID. A targeted mutation of *Taf7l* led to an arrest in spermatogenesis at the end of the round spermatid stage. Transcriptome analysis of *Taf7l*-deficient and WT testis [40] identified some 1,440 genes that were down regulated by more than two-fold, and 726 by over three-fold, in the mutant mice. Furthermore TBL1, whose ablation also leads to a developmental arrest of round spermatids [59], was found to co-occupy active promoters with TAF7L, and the two GTFs were suggested to function together on a subset of postmeiotic genes. Remarkably, none of the genes found to be downregulated in *Taf7l*-deficient testis are also deregulated in RFX2 or CREM deficient testis. This is striking support for the model that there are relatively few master transcriptional regulators for the postmeiotic phase, and that each one controls largely separate groups of genes.

*Gtf2a1l* (ALF) is yet another GTF expressed during spermatogenesis [66,67]. Targeted mutations have not been reported for this gene. *Gtf2a1l* was shown to have a binding site for RFX factors [34], although it was not identified by our ChIP-Seq experiment as being a direct RFX2 target. It’s expression is however reduced slightly more than 2-fold in the *Rfx2* ^*-/-*^ mouse. This reduction may contribute to the broad spectrum of indirectly downregulated genes.

### RFX2 regulates a large cohort of cilia related genes in testis

A strikingly large number of *Rfx2*-regulated genes is associated with the formation and function of cilia and flagella, which are complex organelles that are highly conserved among eukaryotes. In mammals, cilia occur as single non-motile cilia on a wide range of cells, motile monocilia on the embryonic node and multiple motile cilia on various epithelia, whereas flagella are found on sperm cells. The regulation of cilia formation and function by RFX factors traces back to metazoans [10]. As the family expanded through evolution, multiple family members remained associated with this function, including RFX3 and RFX4 in mammals, as well as RFX2 and RFX7 in non-mammalian vertebrates [14,17,18,23,24,26,27]. This study underlines a central role for mouse RFX2 in the regulation of cilia associated genes during spermatogenesis.

RFX proteins control specific subsets of core genes required for cilia formation from *C*. *elegans* to mammals (for review see [11,12]). In particular, RFX factors regulate genes encoding intraflagellar transport (IFT) components and Bardet-Biedl syndrome (BBS) proteins required for building cilia. RFX factors were also shown to regulate transition zone components in *C*. *elegans*, *Drosophila*, *Xenopus* and mice [11,12,68,69]. Transition zone proteins form a specialized structure at the base of the axoneme that connects the distal end of the basal body to the plasma membrane (for review see [70,71]). Whereas many of the IFT genes are indeed bound by RFX2, none are downregulated in *Rfx2* ^*-/-*^ testes at either time point (S1 Table). Many of the *Bbs* genes are also bound by RFX2, but none are downregulated more than 2-fold in *Rfx2* ^*-/-*^ testes. However, expression of some of these genes is significantly decreased by slightly less than the arbitrary 2-fold threshold applied for inclusion in S1 Table. As for IFT and BBS genes, most of the transition zone associated genes are also bound by RFX2 in the testis (*Rpgrip1L*, *B9D1*, *D2*, *Tctn1*, *Tctn2*, *Tctn3*, *Tmem216*, *Tmem138*, *MKS1*, *Tmem17*, *Tmem231*, *Tmem237*, *Cep290*, *Sdccag8*). Only two of these are upregulated at P21 (*Tcnt1)* or downregulated at P30 *(Tmem*231). *Nphp3*, *Nphp4*, and *Nek8* are downregulated in *Rfx2* ^*-/-*^ testis but do not appear to be direct RFX2 targets. Collectively, these results show that RFX2 binds to the promoters of many core genes required for building cilia, but does not seem to play a dominant role in controlling their expression. This implies that other TFs are implicated in regulating genes encoding IFT, BBS and transition zone components in the testis. Other RFX family members expressed in the testis, such as RFX1 or RFX3, are good candidates, as they could function redundantly with RFX2 to regulate core ciliary genes.

Surprisingly, several IFT (IFT74, IFT81) and cilia associated genes are upregulated at P21 in *Rfx2* ^*-/-*^ testes (S9A Fig, S1 Table). This may imply that RFX2 represses these genes during spermatogenesis. However, since RFX factors can function as homo or heterodimers, an alternative hypothesis is that a controlled balance between different dimeric RFX complexes is required for precise tuning of ciliary gene expression. Perturbing this balance by ablating RFX2 may thus generate new RFX complexes capable of activating these upregulated genes.

In contrast to the core ciliary genes, many genes required for ciliary motility are downregulated in *Rfx2* ^*-/-*^ testes. Among 32 genes in which defects lead to primary ciliary dyskinesia, six (*Dnaic2*, *Ccdc40*, *Armc4*, *Ccdc164/DRC1*, *Rsph9*, *Ccdc11*) are strongly downregulated in *Rfx2* ^*-/-*^ testes at both time points, and three others only at P30 (*Ccdc65*, *Zmynd10*, *Ccno)* (for review see [72]). Many genes coding for structural components of flagella or centrioles are also downregulated (S1 Table). RFX2 is thus only critical for the expression of a specific subset of ciliary genes in the testis.

In zebra fish and *Xenopus*, RFX2 has been implicated in the motile cilia differentiation program required for the establishment of left/right asymmetry during embryonic development and the formation of motile cilia in various epithelia [24,26]. We did not observe any major defects in left/right asymmetry or multiciliated epithelia in *Rfx2* ^*-/-*^ mice in two different genetic backgrounds. This is unexpected as *Rfx2* is expressed strongly in the mouse embryonic node and multiciliated epithelia [25,27]. The absence of aberrant left/right or ciliated-epithelia phenotypes in *Rfx2* ^-/-^ mice could be due to partial redundancy between RFX transcription factors. As *Rfx3* ^-/-^ mice display left/right asymmetry defects, it is possible that RFX3 (or other RFX factors) can fully compensate for the deficiency in RFX2, whereas RFX2 cannot compensate efficiently for the loss of RFX3. The opposite seems to be true in the testes, as the few *Rfx3* ^*-/-*^ males that survive past birth do not show defects in sperm or flagella production (B. Durand, personal communication), showing that RFX2 and RFX3 do not have redundant functions in this cell type.

In summary, our study implies that RFX TFs have evolved to regulate specific sets of cilia-related genes in different tissues. RFX2 appears to have become specialized for regulating a specific subset of cilia/flagella-related genes required for spermatid development. Double mutants will need to be studied to further understand how different RFX factors exert their respective functions in different mouse tissues. Furthermore, the complex interplay between members of the RFX family, notably *Rfx2* and *Rfx3*, in the regulation of cilia-related genes in different tissues can now be sorted out genetically using the available conditional mutants.

### *Rfx2* ^-/-^ mice and mutants defective in RNA processing proteins share a similar arrest in spermatid development

Several mutant mice having defects in RNA biogenesis or small-RNA regulated processes show a developmental arrest in spermatogenesis similar to that of *Rfx2* ^-/-^ mice (S4 Table). The genes mutated in these mouse models are not regulated by RFX2. However, the similar phenotype prompted us to examine whether other genes associated with RNA processing and small RNA pathways could be regulated by RFX2. A recent proteomic analysis identified 88 proteins associated with the Chromatoid Body, which is generally believed to be the site of post-nuclear RNA processing and may also contain translationally suppressed mRNAs [73]. Of these proteins, TEKT4 (tectin 4) is encoded by a gene downregulated in *Rfx2* ^*-/-*^ testis at P30. Another recent study demonstrated that many genes required for acrosome or flagella function are downregulated in the *maelstrom* mutant, which carries a mutation in the gene encoding MAEL, a conserved core component of the piRNA pathway [74]. This suggests that RFX2 and MAEL regulated pathways could converge on common genes, and that this might underlie the comparable phenotypes observed in *Rfx2*-deficient mice and piRNA biogenesis pathway mutants. However, we cannot exclude that RFX2 could regulate the expression of small RNAs that our RNA-seq analysis could not reveal. The transcription factor A-MYB has also been shown to regulate piRNA expression in mouse germ cells and many A-MYB target genes have been identified by ChIP-Seq experiments [75]. The binding motif identified in promoters of A-MYB target genes, including piRNA clusters, does not overlap with the well-defined RFX binding motif [75]. In addition, more than 90% of A-MYB targets identified by ChIP-Seq are unique to A-MYB and do not overlap with RFX2 targets. Thus, although A-MYB could regulate RFX2 [33], the two transcription factors do not regulate the same sets of target genes.

### *Rfx2* ^-/-^ mice display polarization defects in post meiotic germ cells

Early in normal spermatid development, a crucial polarity develops such that the acrosome will form and define the anterior end of the sperm, while at the opposite cellular pole, the centriole pair will move to the plasma membrane and initiate growth of the axoneme, which will eventually grow to form the interior elements of the flagellum [43,44]. Failure of normal acrosome cap formation is a characteristic of polarity loss. The mechanisms that set up this polarity are not well understood in spermatids. The PAR3-PAR6-aPKC complex was shown to be required to establish cell polarity [76], but we did not observed any defects of the distribution of these markers (S3 Fig), suggesting that only downstream actors of this complex are likely to be affected in *Rfx2* ^*-/-*^ mice. Cell polarity in many systems is set up by cell junctions with surrounding tissue and involves a set of highly conserved genes. Spermatids are connected to Sertoli cells by adherens-like junctions at this stage [50]. Mutation of junctional adhesion molecule-C (JAM-C) resulted in general polarization failure of spermatids and acrosomic failure similar to that observed in *Rfx2* ^*-/-*^ mice [77]. Around 20 genes required for cell adhesion or cell junctions are downregulated in *Rfx2* ^*-/-*^ testes, which could account for the observed spermatid defect.

Microtubules and associated motors responsible for movement of vesicles and macromolecules are effectors of cell polarization, and required for Golgi organization and acrosome formation in spermatids [47,48,78,79]. 24 genes mapping to Golgi GO terms are downregulated in *Rfx2* ^*-/-*^ testes (S9B Fig) and this may contribute to disruption of overall Golgi organization and acrosome formation in *Rfx2* ^*-/-*^ spermatids. For example, RAB27a/b is known to function as an adapter for acrosome-bound vesicles [46], and *Rab27b* is down regulated ~3-fold. 20 other downregulated genes also have GTPase regulator activity. Furthermore, some 28 microtubule-related genes are downregulated in *Rfx2* ^*-/-*^ testis, which could also contribute to overall polarity and vesicular transport failure (Fig 10C). Hence, defective spermatogenesis in *Rfx2* ^*-/-*^ mice is likely to result from alterations in several different cellular pathways.

#### Concluding remarks

Our results demonstrate that RFX2 is a crucially important TF for orchestrating the postmeiotic phase of sperm development. The morphology of the mutant and characterization of RFX2-regulated pathways may provide a useful basis for assigning genetic causes to human infertility, and ultimately for deciphering the regulatory networks that direct sperm formation.

## Materials and Methods

### Generation of *Rfx2*-deficient mice

The conditional targeting vector (Fig 1A) carried loxP sites inserted into the introns flanking exon 7, which encodes a large segment of the DNA binding domain. Deletion of exon 7 is predicted to alter the reading frame if either exon 5 or 6 is spliced to exon 8 (exon 6 is variably included in the mature transcript). The vector also contained a FRT-flanked neomycin selection cassette. The vector was electroporated into R1 ES cells (strain 129/Sv) by the Mouse Biology Program at University of California, Davis. Targeted clones were identified using PCR and primer sets with outside members located both upstream and downstream of the homology region of the vector (Fig 1B and 1C). Correctly targeted clones were introduced into C57BL/6 blastocysts to generate chimeras. These were bred to C57Bl/6 mice and offspring tested for transmission by PCR analysis of tail biopsy extracts (Fig 1D) using primers described in S5 Table. For initial experiments exon 7 was removed by breeding to a universal Cre-expressing strain, FVB/N-Tg(ACTB-cre)2Mrt/3, and deletion of exon 7 in offspring was verified by PCR (Fig 1D). Heterozygous mice of either gender were fertile and were mated together to generate *Rfx2* ^*-/-*^neo^+^ mice, which were maintained as a mixed background line. Age-matched littermates were used whenever possible. A second line, without the neo cassette, was developed by breeding to a flipase expressing strain, B6;SJL-Tg(ACTFLPe)9205Dym/J [80]. Offspring were selected for loss of the neo cassette and flipase transgene. This strain was backcrossed onto a C57BL/6 genetic background. The phenotype of *Rfx2* ^*-/-*^ animals appears identical with respect to testis morphology for animals from either the C57BL/6 or mixed background lines.

Mice were treated in accordance with the American Veterinary Medicine Association (AVMA) Guidelines for Euthanasia of Laboratory Animals and with French Institutional guidelines in compliance with French and European animal welfare regulations (authorization n° 562, French Ministry of Education and Research). All animal studies were approved by the Institutional Animal Care and Use Committee (IACUC) of the University of South Carolina (AUP No 1818) and by the Ethics Committee of the University of Lyon-1 (BH 2008–01).

### Histology

Animals were sacrificed by CO_2_ anesthesia. Testes were fixed in Bouin’s solution and epididymis in PBS containing 4% paraformaldehyde and processed to produce paraffin sections. Dewaxed sections were stained with hematoxylin and eosin or by PAS using Light Green SF Yellowish as counterstain.

### Western blotting

SDS extraction of testes and Western blotting were carried out as previously described [81]. Separation was in an 8% (RFX2) or 15% gel (TNP1, TNP2). Primary antisera to TNP1 (1:5000) and TNP2 (1:5000) [82], RFX2 (1:250, C-15, Santa Cruz Biotechnology, Santa Cruz, CA) and actin (1:1000, A-2066, Sigma-Aldrich, St Louis, MO) were detected with HRP-conjugated donkey anti-rabbit IgG and anti-goat IgG (1:1000, Jackson ImmunoResearch Laboratories, West Grove, PA) and chemiluminescence [83].

### Reverse transcriptase PCR

For semi-quantitative RT-PCR, total RNA (1 μg) was reverse transcribed using the Superscript III First Strand Synthesis Kit (Invitrogen/Life Technologies) and random hexamer primers according to the manufacturer’s directions in a final volume of 20 μl. After a 1 to 10 dilution, 1 μl was used for a 50 μl PCR reaction. Control reactions lacking reverse transcriptase were done in parallel.

### Electron microscopy

Mice (P25 to P40) were anesthetized by i.p. injection of sodium pentobarbital (0.1 ml/100g b.w.—CEVA santé Animale, France) and fixed by intra-cardiac injection (adapted from [84]) with 2% glutaraldehyde and 0.5% paraformaldehyde in 0.1 M Sodium cacodylate, pH 7.35. Testes were cut into cubes of 1 mm^3^ in fresh fixative solution and left for 2 days at 4°C. After extensive washing in 0.15 M sodium cacodylate (4 x 1h at RT and o/n at 4°C) samples were postfixed in 0.15M sodium cacodylate containing 1% OsO4 for 1 hour at RT, briefly rinsed in distilled water and dehydrated through a graded series of ethanol solutions (from 30° to 100°) and two baths of propylene oxide for 15 min each. After substitution and impregnation, small blocks were embedded in epoxy resin in flat silicon molds and polymerized at 56°C for 48h. Ultrathin sections were cut with a UC7 Leica ultramicrotome. Ultrathin sections were contrasted in aqueous uranyl-acetate and lead citrate solutions using a Leica ultrostainer. Sections were observed with a Philips CM120 transmission electron microscope at 120Kv.

### Immunohistochemical analysis

Testes were isolated from *Rfx2* ^*+/+*^ and *Rfx2* ^*-/-*^ mice (P30 to P40), fixed o/n in 4% PFA, embedded and frozen in 15% sucrose, 7.5% gelatin. Immunofluorescence and apoptosis experiments were performed with 14 μm cryosections. Briefly, samples were permeabilized in 0.1% Triton for 5 min, and nonspecific antibody binding was blocked with 3% BSA, 10% FBS. Primary antibodies were incubated o/n at 4°C. Secondary antibodies (anti-rabbit Alexa 488 or anti-mouse Alexa 594 or Alexa 555, Invitrogen, both at 1:500) were incubated for 2 h at RT. Nuclei were counterstained with DRAQ5. Fluorescence was observed under a LSM 510 Confocal microscope. Primary antibodies were: anti mouse H2AX (1/500, Millipore), Acetylated-tubulin (1/1000, T6793 Sigma), Beta-catenin (1/50, C2206 Sigma), ZO-1 (1/25, PA5-28858 Zymed), PAR3 (1/100, 07–330 Millipore), PAR6 (1/50, sc-67393 Santa Cruz Biotechnology), aPKC (1/400, sc-216 Santa Cruz Biotechnology). PAR6 and aPKC antibodies were kind gifts from M. Montcouquiol.

#### Acrosomes

Testes were fixed in 4% paraformaldehyde in PBS overnight, incubated in 30% sucrose, embedded in sucrose/gelatin and cut with a cryostat. Frozen sections (20 μm) were washed in PBS and incubated 2 hours at RT in PBS with Fluorescein isothiocyanate-conjugated peanut agglutinin (FITC-PNA, Sigma L7381) at a final concentration of 20 μg/ml. Nuclei were counterstained with Hoechst. Sections were washed twice in PBS and mounted in Dako fluorescence mounting medium (Dako S3023) and observed with a 63X/1.4 oil objective on a Leica SP5 spectral confocal.

#### Apoptosis

Apoptotic cells were visualized according to the TUNEL assay protocol from Roche Diagnostics (Roche 11684795910). 20 μm thick frozen sections (as prepared above) were washed in PBS, permeabilized 2 min at 4°C with 0.1% Triton X-100 in 0.1% sodium citrate freshly prepared, washed twice in PBS 1X and incubated in the TUNEL label solution for 60 min at 37°C, washed twice in PBS and mounted and observed as described above.

#### TNP2

Decapsulated testes were fixed in 4% paraformaldehyde in PBS overnight at 4°C and embedded in paraffin. Sections were mounted on Superfrost plus slides (Fisher Thermo Scientific), rehydrated and subjected to antigen retrieval by microwave heating as described previously [81]. Sections were blocked in PBS containing 0.1% Triton and 10% donkey serum for 30 min at room temp and then exposed to the primary antibody overnight at 4°C. After washing 3X with PBS, 0.1% TritonX-100 slides were exposed to the secondary antibody for 30–45 min at room temp (biotinylated IgG, 1:200, Jackson ImmunoResearch), washed briefly, exposed to Cy2 or Cy3 conjugated streptavidin (1:1000, Jackson) for 15 min at RT, washed, and cover-slipped using Vectashield containing DAPI (1 μg/ml). Slides were examined using an Olympus B41 microscope and digital images recorded with an Olympus DP71 camera. Images were processed using Photoshop.

#### RNA Seq and bioinformatics

See supplementary material.

## Supporting Information

S1 Fig Neither apoptosis nor DNA strand breaks are increased in *Rfx2* ^*-/-*^ testes. TUNEL staining showed no significance difference between numbers of positive-stained cells (arrowheads) in *Rfx2* ^*+/+*^ and *Rfx2* ^*-/-*^ testes (A, B). DNA breaks detected by fluorescent antibody staining of phosphorylated histone H2AX showed no increase in *Rfx2* ^*-/-*^ testes (C). Phosphorylated H2AX staining is normally increased in early spermatocytes associated with the initiation of meiotic recombination (arrowheads), and at the inactive X-Y chromosome pair during pachytene (arrows).
(TIF)

S2 Fig Aberrant expression of transition protein 2 (TNP2) in arrested round spermatids of *Rfx2* ^*-/-*^ testes. (A) The expression of TNP1 or TNP2 was assessed by western blotting in testis extracts from *Rfx2* ^*+/+*^ and *Rfx2* ^*-/-*^ mice of 30 and 104 days of age. Whereas TNP2 is normally expressed, TNP1 is never present in *Rfx2* ^*-/-*^ testes showing that differentiation is blocked before TNP1 expression. (B) TNP2 protein (red, anti TNP2 antibody) is normally detected only in the nuclei (blue, Dapi) of spermatids that have begun the process of chromatin condensation, beginning about step 10, and TNP2 staining is found prominently over moderately condensed nuclei in WT testes. (C) In mutant testes, where no cells develop to the point at which TNP2 is first detected in the normal situation, prominent TNP2 staining was observed largely over the cytoplasm of arrested nuclei present in multinucleated giant cells.
(TIF)

S3 Fig No flagella protrude into the lumen of seminiferous tubule, but polarity markers are correctly distributed before the round spermatid stage, in *Rfx2* ^*-/-*^ testes. (A) Testis sections of 40 days old males were stained for acetylated tubulin (red), acrosomes (peanut-agglutinin, green) and nuclei (Dapi). Whereas bundles of flagella extend in the lumen of seminiferous tubule sections in control testes (white arrows), such bundles where not observed in *Rfx2* ^*-/-*^ testes. Strong acetylated tubulin staining was seen inside round spermatid syncitia (arrowheads), highlighting the particular microtubule network organization in these cells. (B) Testis sections of 40 days old males were stained for several polarity markers. Beta-catenin, ZO1, PAR6 and PAR3 are enriched at the BTB in both control and *Rfx2* ^*-/-*^ seminiferous tubules (arrows). In *Rfx2* ^*-/-*^ testes, all markers are also enriched at the periphery of the symplasts (arrowheads). aPKC is expressed in elongating spermatids in WT testes (arrows) as well as in arrested symplasts (arrows), illustrating that aPKC expression is induced but that spermatids do not elongate.
(TIF)

S4 Fig Scatter plot representing the fold-change in expression in *Rfx2* ^-/-^ relative to *Rfx2* ^+/+^ testis as a function of mean normalized expression. Red dots indicate significantly (p<0.01) altered expression.
(TIF)

S5 Fig Heatmap representation of cluster analysis of DE expressed genes for enriched Transcription factor binding sites obtained from the Jaspar database [1]. RFX1, 2 and 5 binding motifs are highly enriched in genes up-regulated at P21 and in genes down-regulated at P30 or at both P30 and P21.
(TIF)

S6 Fig Peaks obtained in each ChIP-Seq experiment are shown for 4 representative genes. Input DNA was used as background control. *Dync2Li1* was selected as a control gene because it is a known target of RFX factors in various other cell types. The remaining three genes are involved in ciliogenesis and are downregulated in *Rfx2* ^-/-^ testes at both P21 and P30.
(TIF)

S7 Fig Gene ontology (GO) analysis of RFX2 target genes identified by ChIP-Seq. Histograms represent the number of genes matching each significantly enriched GO term (biological process or cellular component) in the RFX2 ChIP-Seq gene list. Statistical significance (p-value) is provided for each GO term.
(TIF)

S8 Fig Gene ontology (GO) analysis of DE genes at P21 and P30 in *Rfx2* ^*-/-*^ versus *Rfx2* ^*+/+*^ testes. Histograms represent the number of genes matching each significantly enriched GO term (biological process or cellular component). Statistical significance (p-value) is provided for each GO term.
(TIF)

S9 Fig DE genes in *Rfx2* ^-/-^ testis associated with cilia or golgi. (A) Bars show the expression levels (RPKM, reads per Kb per million) in *Rfx2* ^*+/+*^ (grey) and *Rfx2* ^*-/-*^ (black) testis for genes that are upregulated genes at P21 and are included in the Syscilia Gold or Potential lists. RFX2 targets are indicated above the bars. Genes are ordered according to their expression level in WT mice. (B) P30 downregulated genes assigned to golgi-related GO terms. Expression in *Rfx2* ^-/-^ testis is expressed relative to *Rfx2* ^+/+^ testis. RFX2 targets are indicated above the bars.
(TIF)

S10 Fig RFX2 and CREM regulate distinct sets of genes. (A) Venn diagram show overlaps between genes that are downregulated >2-fold in *Rfx2* ^-/-^ testis at P21 (top), downregulated >2-fold in *Rfx2* ^-/-^ testis at P30 (middle) or constitute direct RFX2 targets (bottom), and genes that are downregulated >2-fold in *Crem* ^-/-^ testis [2] or are CREM-regulated genes that are likely to be direct targets in male germ cells [2,3]. (B) Developmental expression profiles derived from Laiho et al 2013 [4] are shown for RFX2 target genes that are downregulated significantly (p<0.001) by >2X in *Rfx2* ^-/-^ testis at P21 (top), in *Rfx2* ^-/-^ testis at P30 (middle) or in *Crem* ^-/-^ testis according to Kosir et al 2012 (bottom).
(TIF)

S1 Table Summary of RFX2 target genes and differentially expressed genes in Rfx2-/- testes at P21 and P30. (XLSX)

S2 Table Comparison between RFX2 and CREM regulated genes. (XLSX)

S3 Table GO analysis of RFX2 target genes. (XLSX)

S4 Table Mouse mutations leading to an arrest in spermatid development before step 8. (DOC)

S5 Table PCR Primers used to analyze the targeted *Rfx2* locus. (DOC)

S1 Text Supplementary methods and references. (DOCX)
