# Supplementary figures and images for: RFX2 Is a Major Transcriptional Regulator of Spermiogenesis
Source: PLoS Genet. 2015 Jul 10;11(7):e1005368. doi: 10.1371/journal.pgen.1005368 (PMC4498915; doi:10.1371/journal.pgen.1005368)

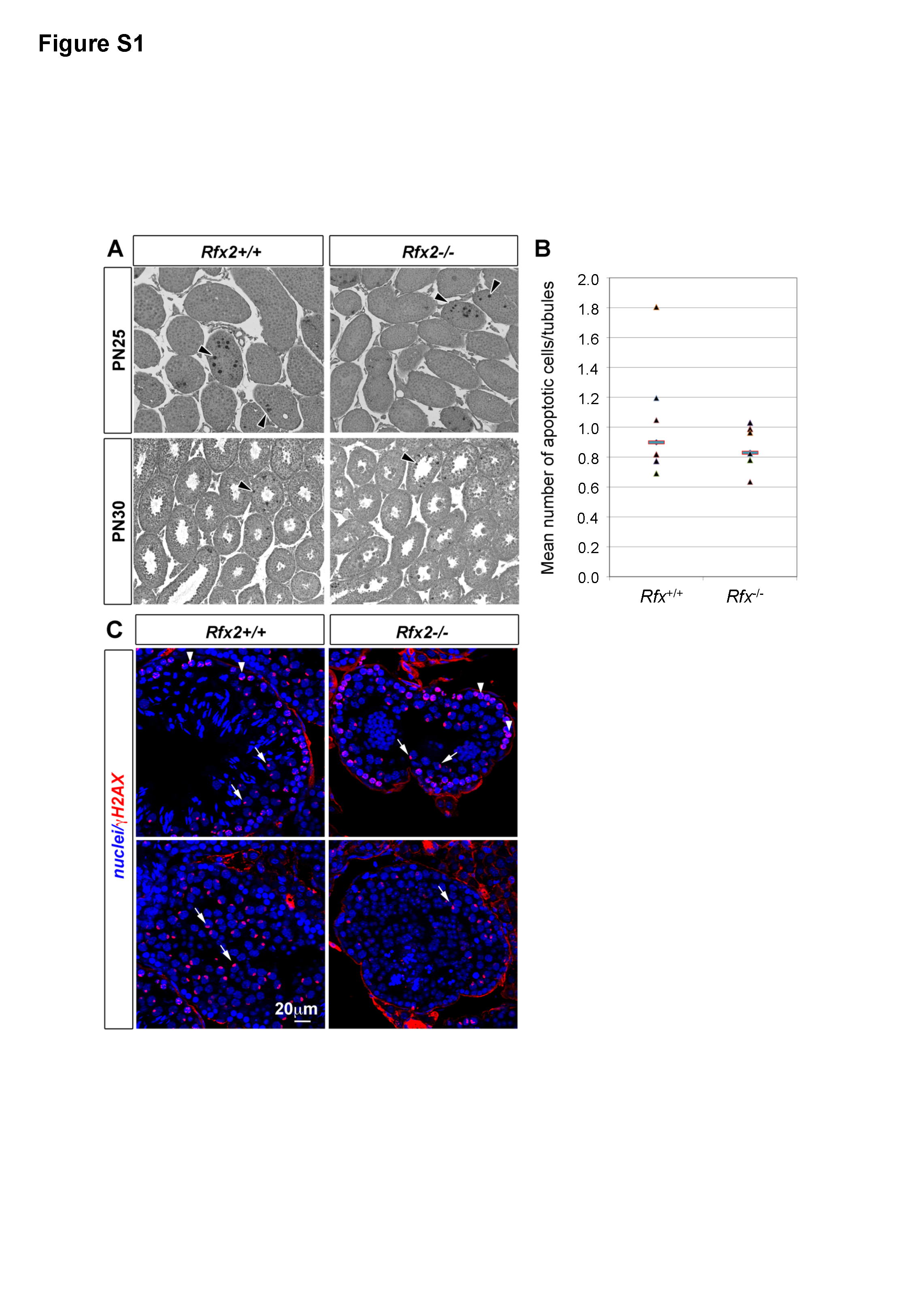

Supplement: S1 Fig — TUNEL staining showed no significance difference between numbers of positive-stained cells (arrowheads) in Rfx2 +/+ and Rfx2 -/- testes (A, B). DNA breaks detected by fluorescent antibody staining of phosphorylated histone H2AX showed no increase in Rfx2 -/- testes (C). Phosphorylated H2AX staining is normally increased in early spermatocytes associated with the initiation of meiotic recombination (arrowheads), and at the inactive X-Y chromosome pair during pachytene (arrows). (TIF) [file pgen.1005368.s001.tif]

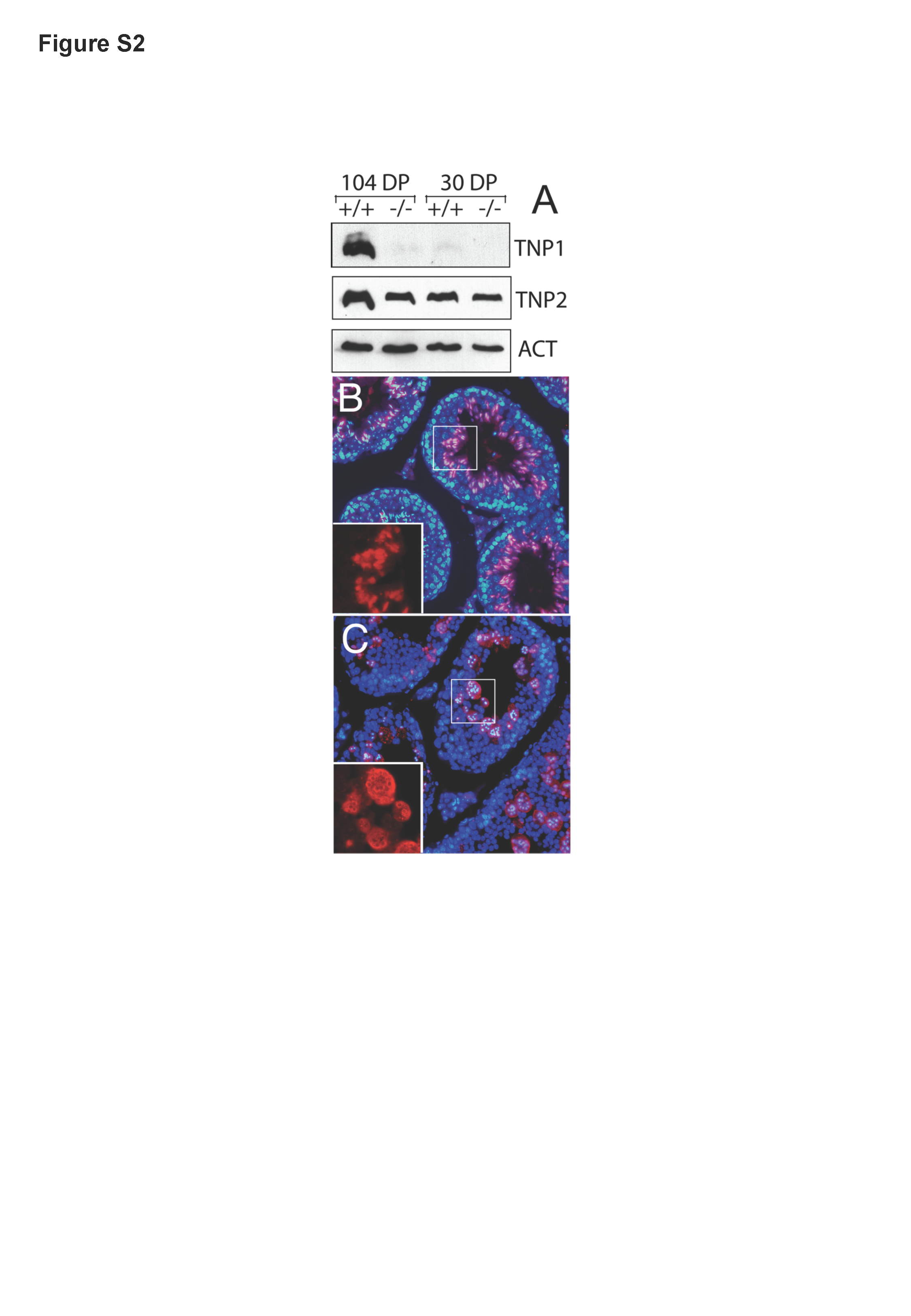

Supplement: S2 Fig — (A) The expression of TNP1 or TNP2 was assessed by western blotting in testis extracts from Rfx2 +/+ and Rfx2 -/- mice of 30 and 104 days of age. Whereas TNP2 is normally expressed, TNP1 is never present in Rfx2 -/- testes showing that differentiation is blocked before TNP1 expression. (B) TNP2 protein (red, anti TNP2 antibody) is normally detected only in the nuclei (blue, Dapi) of spermatids that have begun the process of chromatin condensation, beginning about step 10, and TNP2 staining is found prominently over moderately condensed nuclei in WT testes. (C) In mutant testes, where no cells develop to the point at which TNP2 is first detected in the normal situation, prominent TNP2 staining was observed largely over the cytoplasm of arrested nuclei present in multinucleated giant cells. (TIF) [file pgen.1005368.s002.tif]

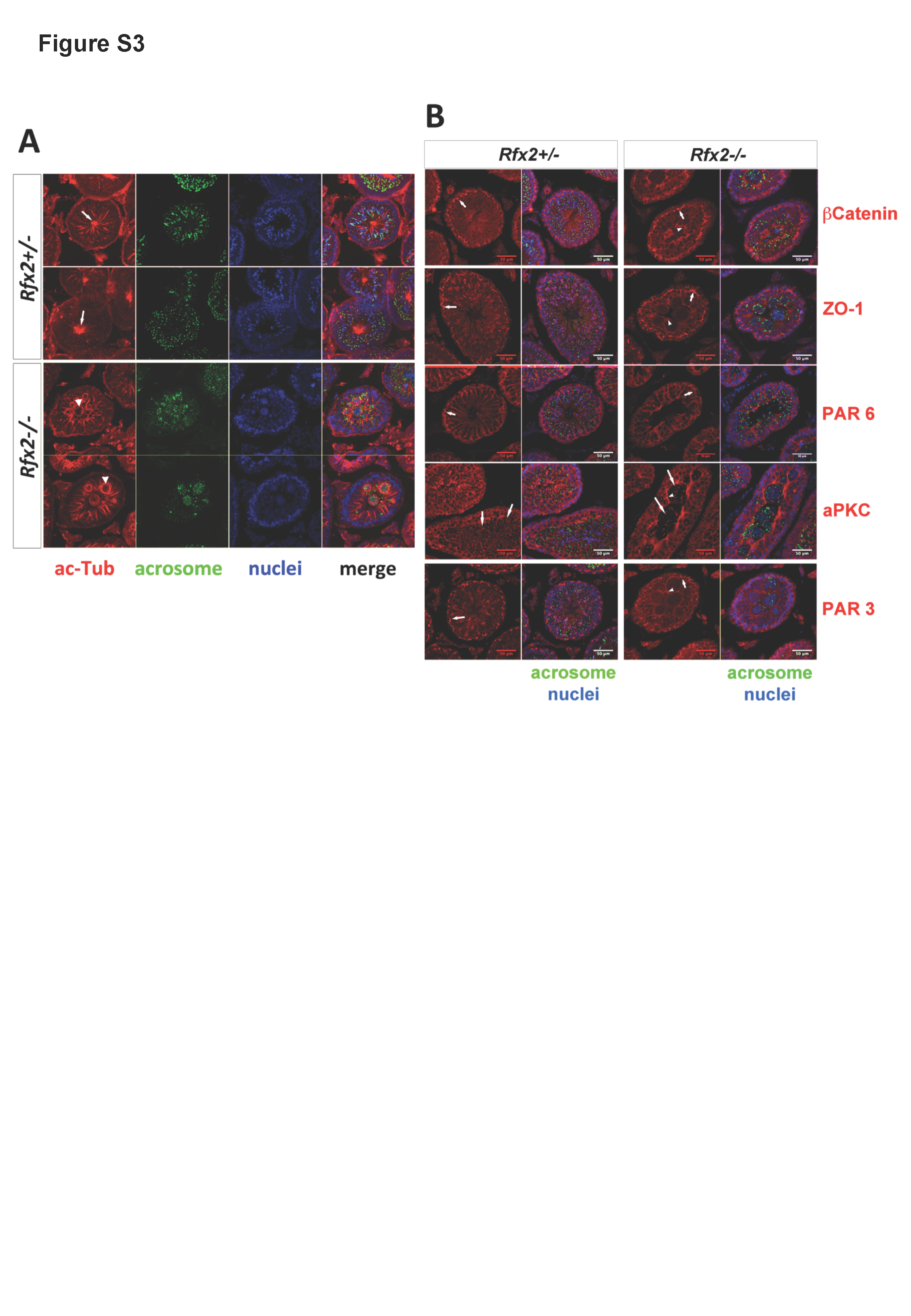

Supplement: S3 Fig — (A) Testis sections of 40 days old males were stained for acetylated tubulin (red), acrosomes (peanut-agglutinin, green) and nuclei (Dapi). Whereas bundles of flagella extend in the lumen of seminiferous tubule sections in control testes (white arrows), such bundles where not observed in Rfx2 -/- testes. Strong acetylated tubulin staining was seen inside round spermatid syncitia (arrowheads), highlighting the particular microtubule network organization in these cells. (B) Testis sections of 40 days old males were stained for several polarity markers. Beta-catenin, ZO1, PAR6 and PAR3 are enriched at the BTB in both control and Rfx2 -/- seminiferous tubules (arrows). In Rfx2 -/- testes, all markers are also enriched at the periphery of the symplasts (arrowheads). aPKC is expressed in elongating spermatids in WT testes (arrows) as well as in arrested symplasts (arrows), illustrating that aPKC expression is induced but that spermatids do not elongate. (TIF) [file pgen.1005368.s003.tif]

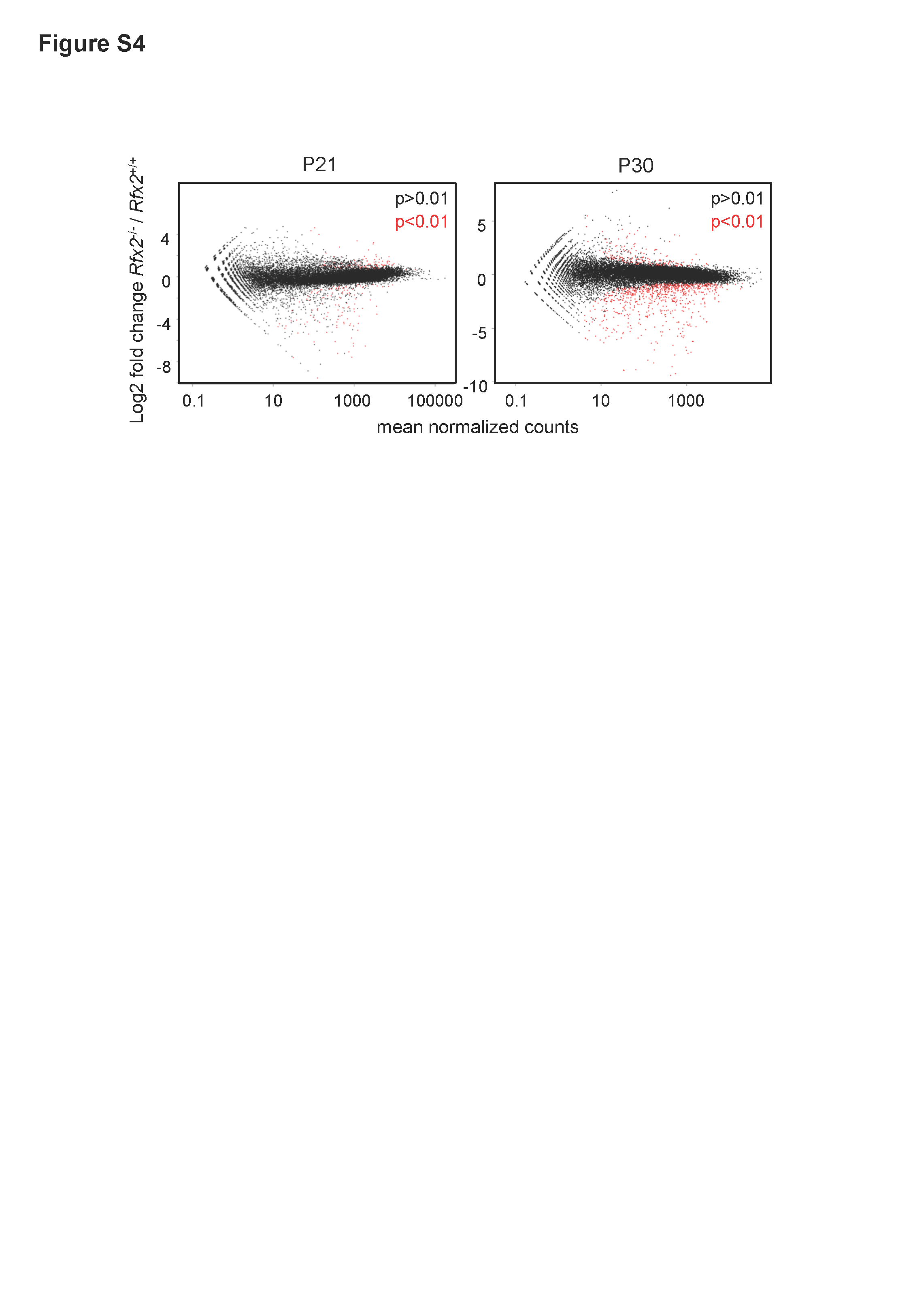

Supplement: S4 Fig — Red dots indicate significantly (p<0.01) altered expression. (TIF) [file pgen.1005368.s004.tif]

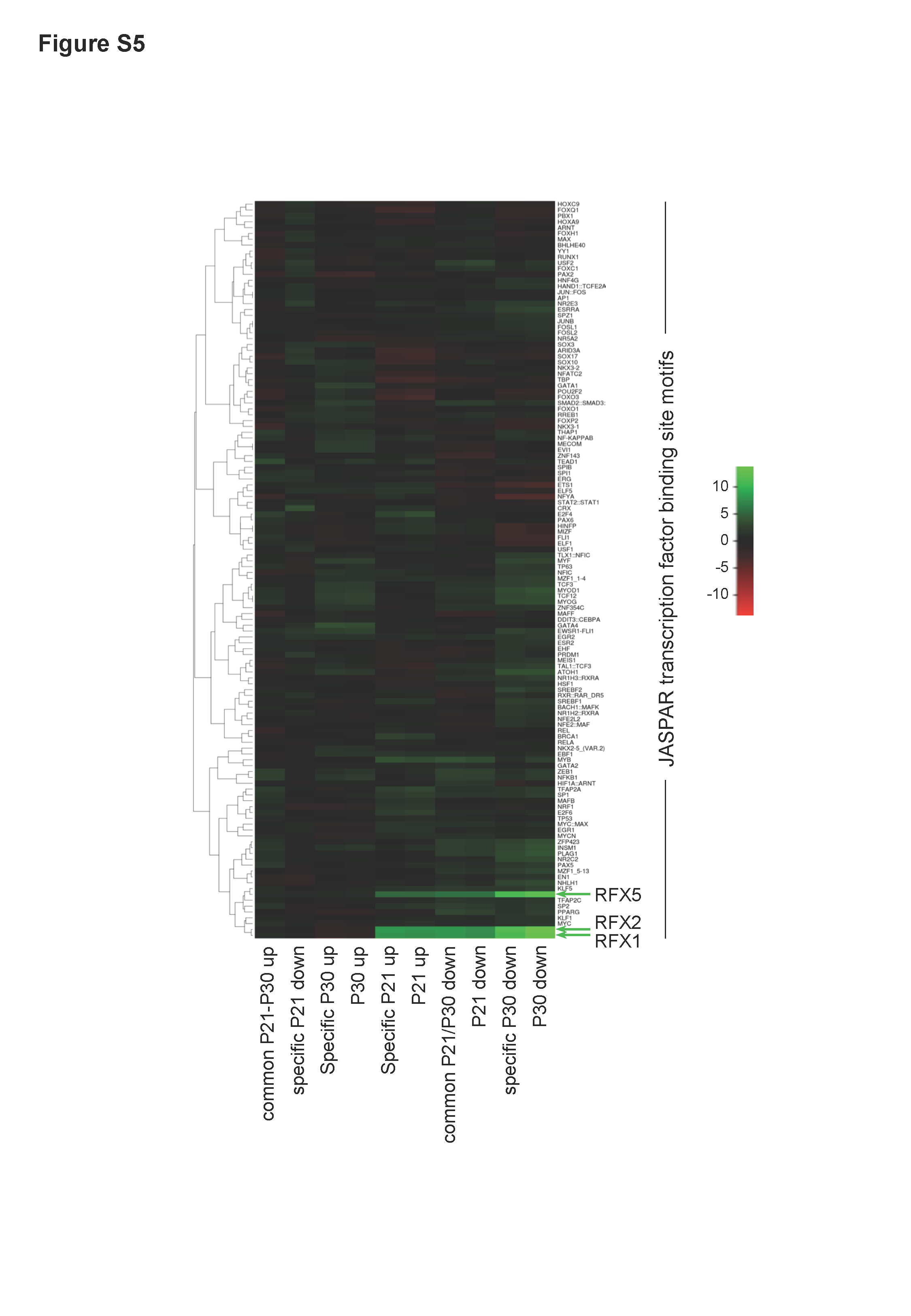

Supplement: S5 Fig — RFX1, 2 and 5 binding motifs are highly enriched in genes up-regulated at P21 and in genes down-regulated at P30 or at both P30 and P21. (TIF) [file pgen.1005368.s005.tif]

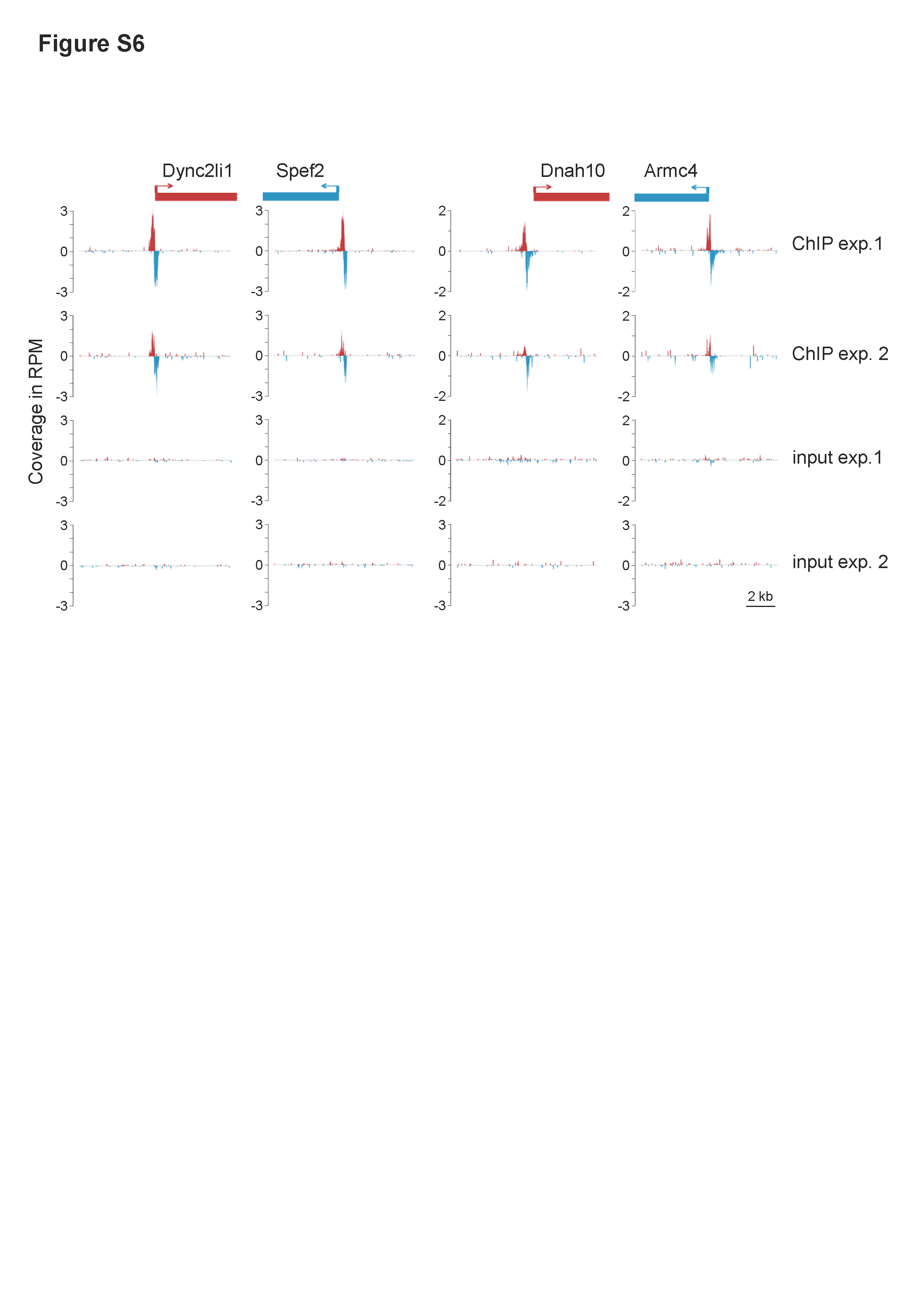

Supplement: S6 Fig — Input DNA was used as background control. Dync2Li1 was selected as a control gene because it is a known target of RFX factors in various other cell types. The remaining three genes are involved in ciliogenesis and are downregulated in Rfx2 -/- testes at both P21 and P30. (TIF) [file pgen.1005368.s006.tif]

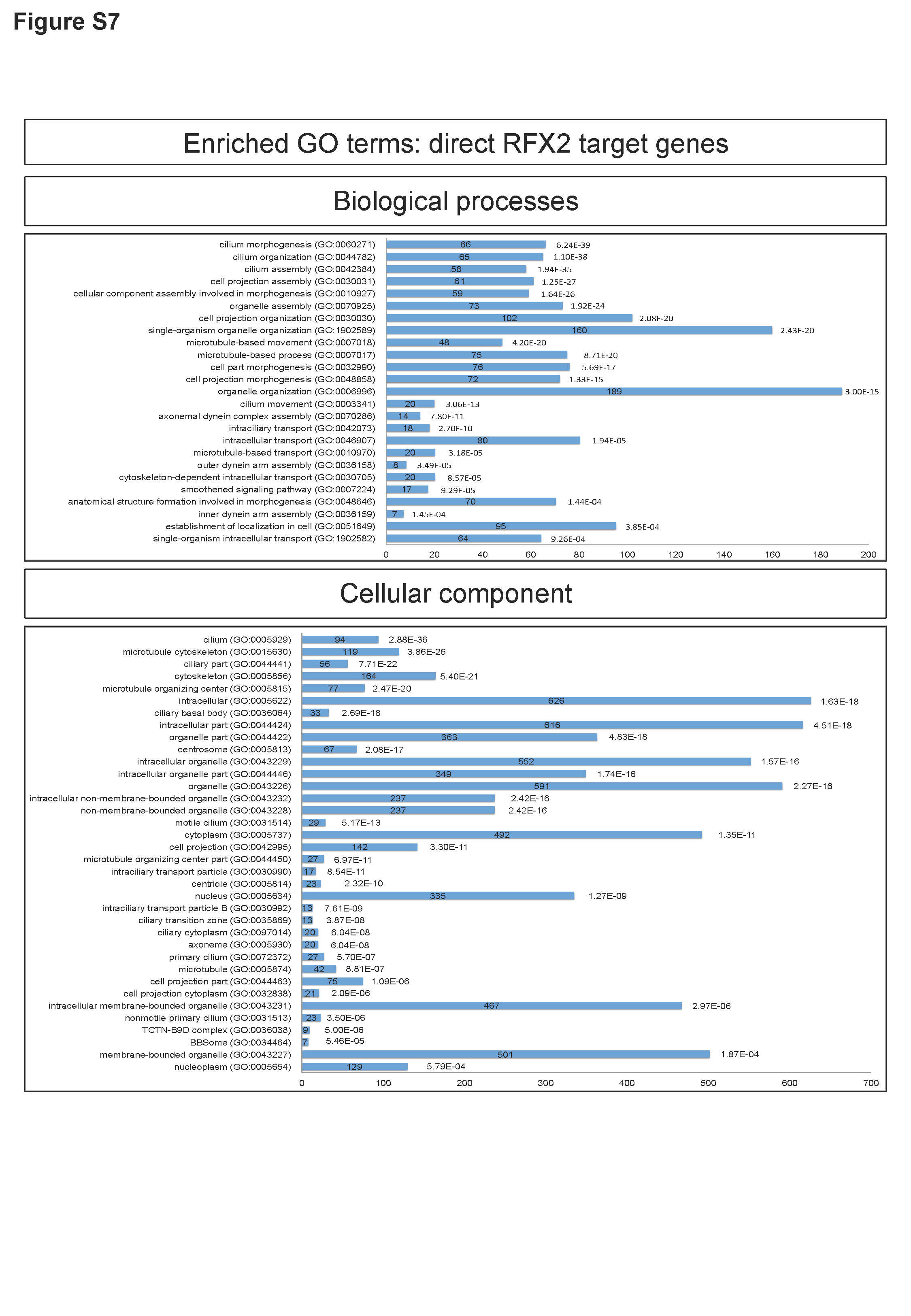

Supplement: S7 Fig — Histograms represent the number of genes matching each significantly enriched GO term (biological process or cellular component) in the RFX2 ChIP-Seq gene list. Statistical significance (p-value) is provided for each GO term. (TIF) [file pgen.1005368.s007.tif]

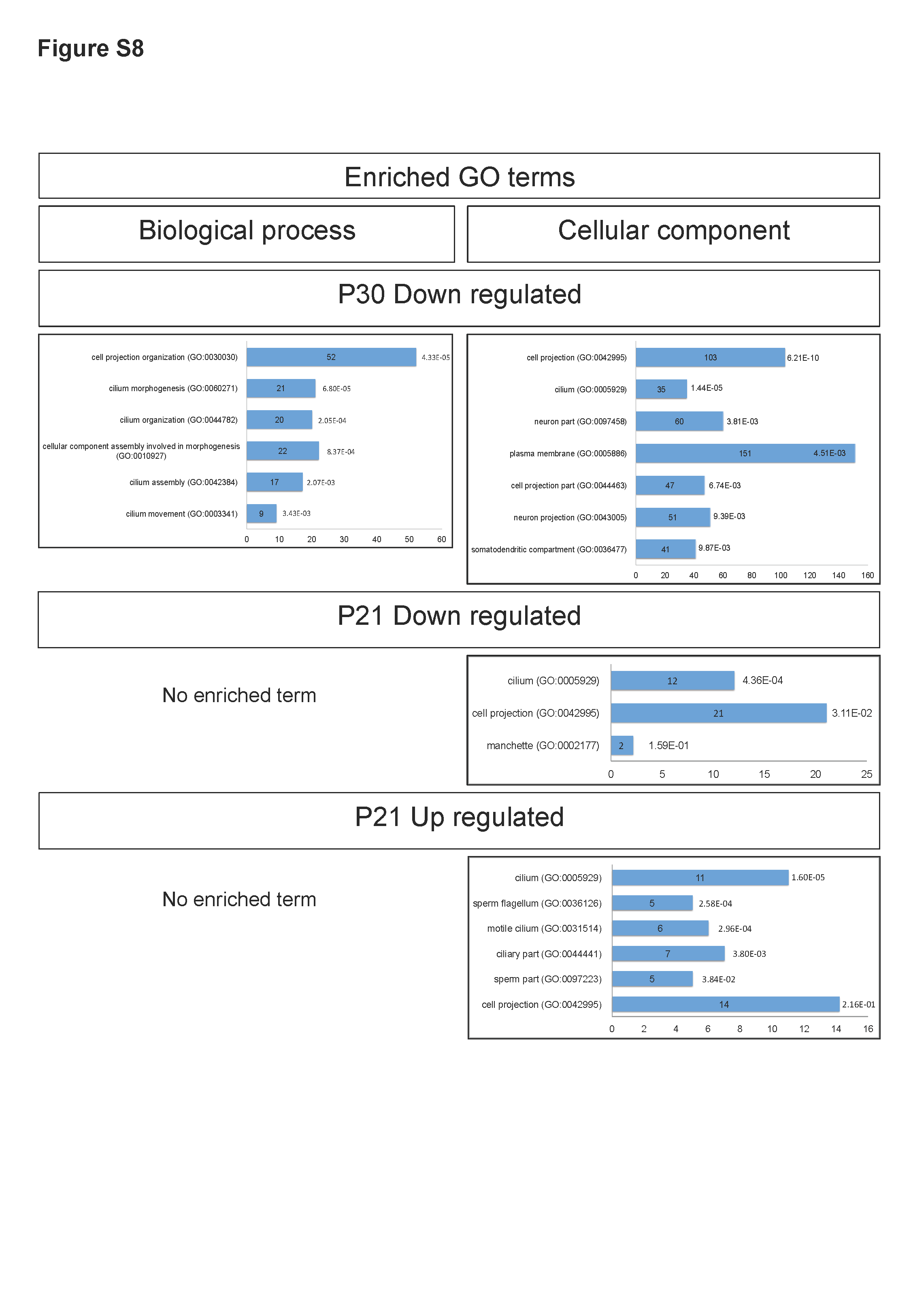

Supplement: S8 Fig — Histograms represent the number of genes matching each significantly enriched GO term (biological process or cellular component). Statistical significance (p-value) is provided for each GO term. (TIF) [file pgen.1005368.s008.tif]

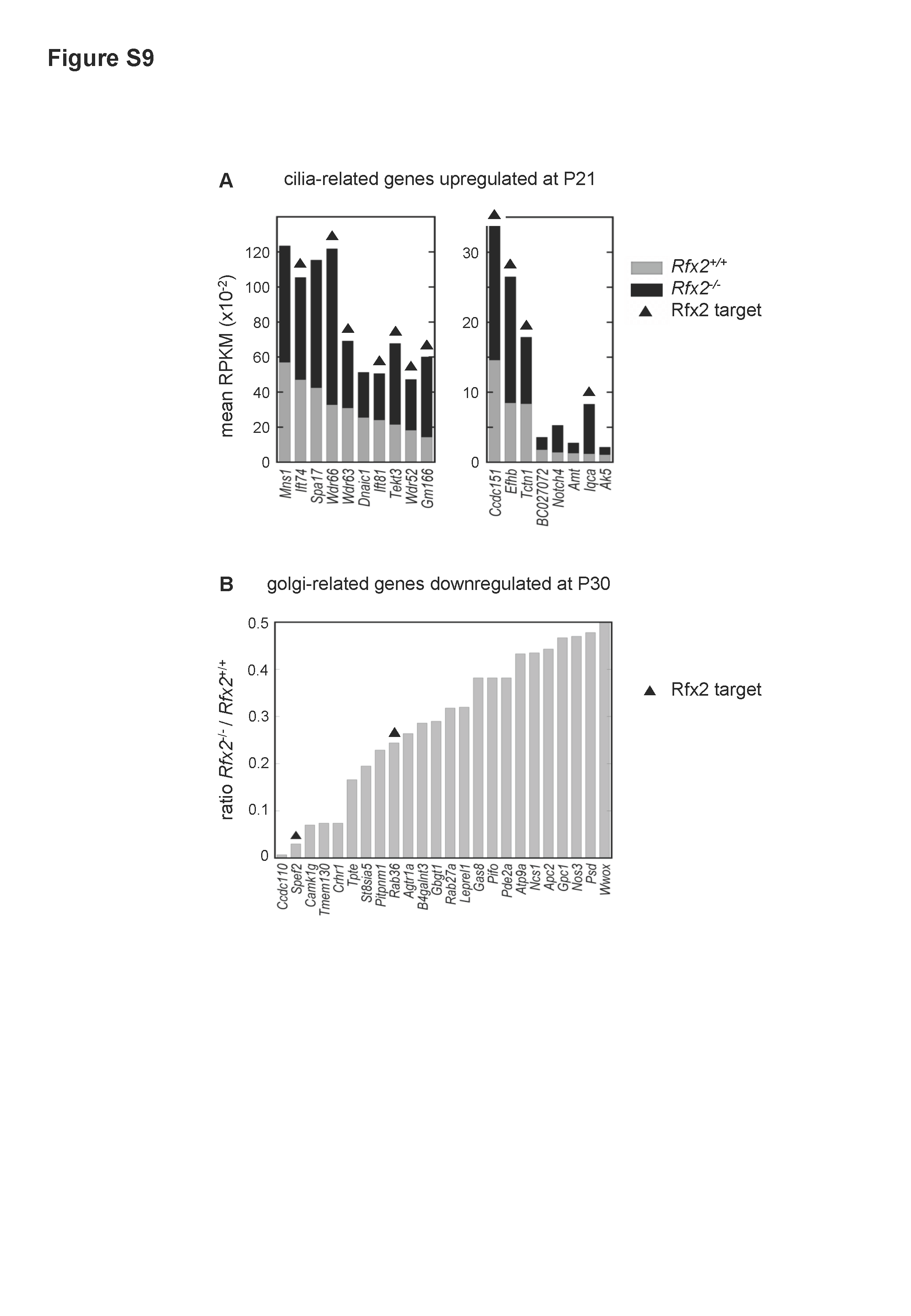

Supplement: S9 Fig — (A) Bars show the expression levels (RPKM, reads per Kb per million) in Rfx2 +/+ (grey) and Rfx2 -/- (black) testis for genes that are upregulated genes at P21 and are included in the Syscilia Gold or Potential lists. RFX2 targets are indicated above the bars. Genes are ordered according to their expression level in WT mice. (B) P30 downregulated genes assigned to golgi-related GO terms. Expression in Rfx2 -/- testis is expressed relative to Rfx2 +/+ testis. RFX2 targets are indicated above the bars. (TIF) [file pgen.1005368.s009.tif]

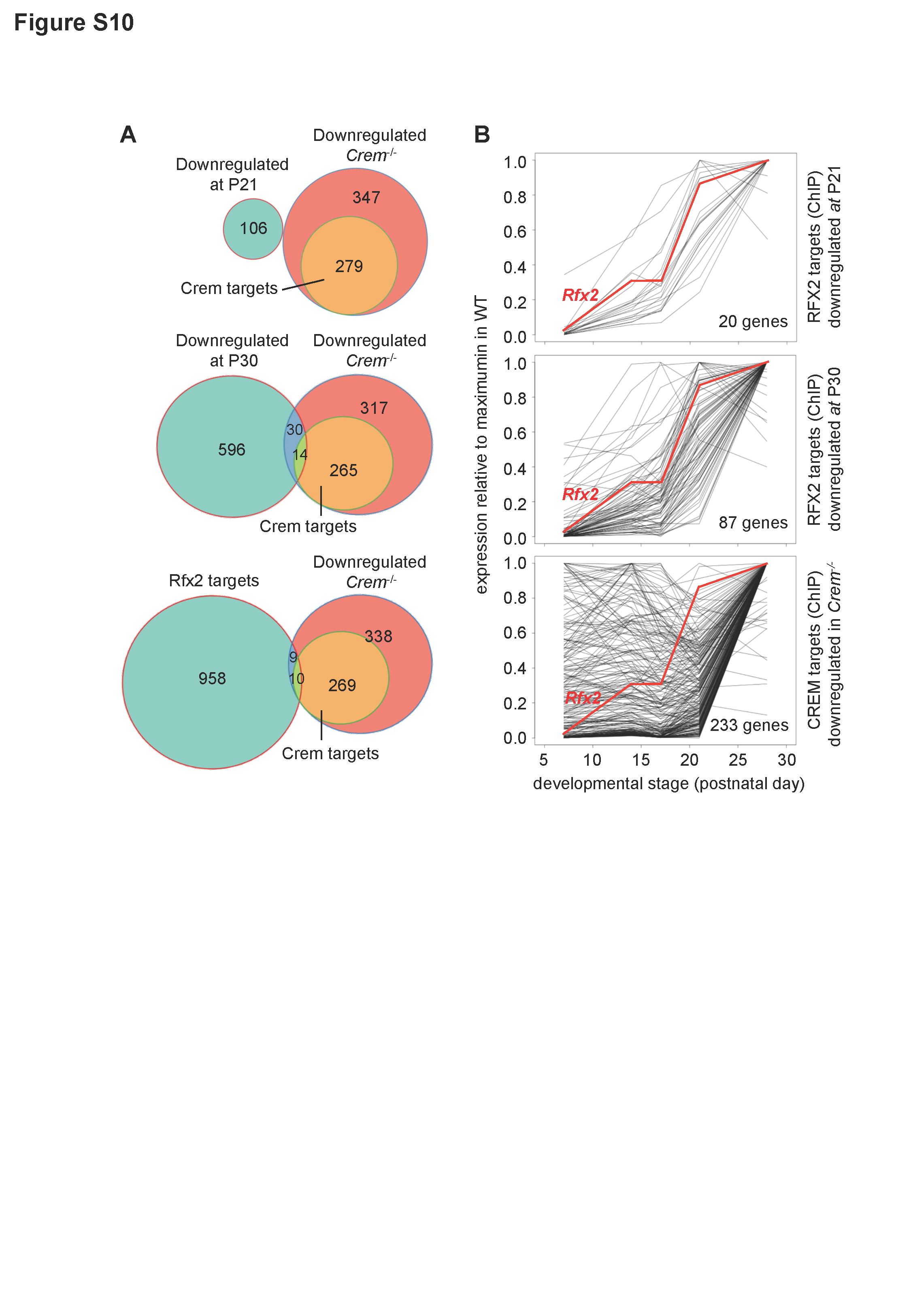

Supplement: S10 Fig — (A) Venn diagram show overlaps between genes that are downregulated >2-fold in Rfx2 -/- testis at P21 (top), downregulated >2-fold in Rfx2 -/- testis at P30 (middle) or constitute direct RFX2 targets (bottom), and genes that are downregulated >2-fold in Crem -/- testis [2] or are CREM-regulated genes that are likely to be direct targets in male germ cells [2,3]. (B) Developmental expression profiles derived from Laiho et al 2013 [4] are shown for RFX2 target genes that are downregulated significantly (p<0.001) by >2X in Rfx2 -/- testis at P21 (top), in Rfx2 -/- testis at P30 (middle) or in Crem -/- testis according to Kosir et al 2012 (bottom). (TIF) [file pgen.1005368.s010.tif]
