# Supplementary material for: RFX2 Is a Major Transcriptional Regulator of Spermiogenesis
Source: PLoS Genet. 2015 Jul 10;11(7):e1005368. doi: 10.1371/journal.pgen.1005368 (PMC4498915; doi:10.1371/journal.pgen.1005368)
Supplement: S4 Table — (DOC) [file pgen.1005368.s014.doc]

**Table S3**. Mouse mutations leading to an arrest in spermatid development before step 8.

| **Functional Category and *genes***^1^ | **Reference** |
| --- | --- |
| ***Transcription*** |  |
| *Crem* | [1,2] |
| *Tbpl1* (Trf2) | [3] |
| *Taf7l* (Taf2q) | [4] |
| ***RNA Processing/regulation*** |  |
| *Boll* (boule-like) | [5] |
| *Celf1* (CUGBP) | [6] |
| *Ddx25* (GRTH) | [7] |
| *Mael* | [8] |
| *Papolb* (TPAP) | [9] |
| *Piwil1* (MIWI) | [10] |
| *Tdrd1* | [11] |
| *Tdrd5* | [12] |
| *Tdrd6* | [13] |
| *Tdrd7* | [14] |
| *Ybx2* (MSY2) | [15] |
| ***Organelle/Junctional components*** |  |
| *Bsg* (Basigin) | [16] |
| *Cadm1* (TSLC1) | [17] |
| *Fndc3a* (sys mutant) | [18] |
| *Mgat1* | [19] |
| *Ranbp1* (Htf9a) | [20] |

^1^Gene names: http://www.informatics.jax.org

1. Blendy JA, Kaestner KH, Weinbauer GF, Nieschlag E, Schütz G (1996) Severe impairment of spermatogenesis in mice lacking the CREM gene. Nature 380: 162-165.
2. Nantel F, Monaco L, Foulkes NS, Masquilier D, LeMeur M, et al. (1996) Spermiogenesis deficiency and germ-cell apoptosis in CREM-mutant mice. Nature 380: 159-162.
3. Martianov I, Fimia GM, Dierich A, Parvinen M, Sassone-Corsi P, et al. (2001) Late arrest of spermiogenesis and germ cell apoptosis in mice lacking the TBP-like TLF/TRF2 gene. Mol Cell 7: 509-515.
4. Zhou H, Grubisic I, Zheng K, He Y, Wang PJ, et al. (2013) Taf7l cooperates with Trf2 to regulate spermiogenesis. Proc Natl Acad Sci USA 110: 16886-16891.
5. VanGompel MJW, Xu EY (2010) A novel requirement in mammalian spermatid differentiation for the DAZ-family protein Boule. Hum Mol Genet 19: 2360-2369.
6. Kress C, Gautier-Courteille C, Osborne HB, Babinet C, Paillard L (2007) Inactivation of CUG-BP1/CELF1 causes growth, viability, and spermatogenesis defects in mice. Mol Cell Biol 27: 1146-1157.
7. Tsai-Morris C-H, Sheng Y, Lee E, Lei K-J, Dufau ML (2004) Gonadotropin-regulated testicular RNA helicase (GRTH/Ddx25) is essential for spermatid development and completion of spermatogenesis. Proc Natl Acad Sci USA 101: 6373-6378.
8. Castañeda J, Genzor P, van der Heijden GW, Sarkeshik A, Yates JR 3rd, et al., (2014) Reduced pachytene piRNAs and translation underlie spermiogenic arrest in Maelstrom mutant mice. EMBO J 33:1999-2019.
9. Kashiwabara S-I, Noguchi J, Zhuang T, Ohmura K, Honda A, et al. (2002) Regulation of spermatogenesis by testis-specific, cytoplasmic poly(A) polymerase TPAP. Science 298: 1999-2002.
10. Deng W, Lin H (2002) Miwi, a murine homolog of piwi, encodes a cytoplasmic protein essential for spermatogenesis. Dev Cell 2: 819-830.
11. Chuma S, Hosokawa M, Kitamura K, Kasai S, Fujioka M et al., (2006) Tdrd1/Mtr-1, a tudor-related gene, is essential for male germ-cell differentiation and nuage/germinal granule formation in mice. Proc Natl Acad Sci USA 103:15894-15899.
12. Yabuta Y, Ohta H, Abe T, Kurimoto K, Chuma S, et al. (2011) TDRD5 is required for retrotransposon silencing, chromatoid body assembly, and spermiogenesis in mice. J Cell Biol 192: 781-795.
13. Vasileva A, Tiedau D, Firooznia A, Müller-Reichert T, Jessberger R (2009) Tdrd6 is required for spermiogenesis, chromatoid body architecture, and regulation of miRNA expression. Curr Biol 19:630-639.
14. Tanaka T, Hosokawa M, Vagin VV, Reuter M, Hayashi E et al. (2011) *Tudor domain containing 7* (*Tdrd7*) is essential for dynamic ribonucleoprotein (RNP) remodeling of chromatoid bodies during spermatogenesis. Proc Natl Acad Sci USA 108:10579-10584.
15. Yang J, Medvedev S, Yu J, Tang LC, Agno JE, et al. (2005) Absence of the DNA-/RNA-binding protein MSY2 results in male and female infertility. Proc Natl Acad Sci USA 102: 5755-5760.
16. Bi JB, Li Y, Sun F, Saalbach A, Klein C, et al. (2013) Basigin null mutant mice are sterile nd exhibit impaired interactions between germ cells and Sertoli cells. Dev Biol 380: 145-156.
17. van der Weyden L, Arends MJ, Chausiaux OE, Ellis PJ, Lange UC, et al. (2006) Loss of TSLC1 causes male infertility due to a defect at the spermatid stage of spermatogenesis. Mol Cell Biol 26: 3595-3609.
18. Obholz KL, Akopyan A, Waymire KG, MacGregor GR (2006) FNDC3A is required for adhesion between spermatids and Sertoli cells. Dev Biol 298: 498-513.
19. Batista F, Lu L, Williams SA, Stanley P (2012) Complex N-Glycans are essential, but core 1 and 2 mucin O-glycans, O-fucose glycans, and NOTCH1 are dispensible, for mammalian spermatogenesis. Biol Reprod 86: 1-12.
20. Nagai M, Moriyama T, Mehmood R, Tokuhiro K, Ikawa M, et al. (2011) Mice lacking Ran binding protein 1 are viable and show male infertility. FEBS Letters 585: 791-796.
