# Supplementary material for: RFX2 Is a Major Transcriptional Regulator of Spermiogenesis
Source: PLoS Genet. 2015 Jul 10;11(7):e1005368. doi: 10.1371/journal.pgen.1005368 (PMC4498915; doi:10.1371/journal.pgen.1005368)
Supplement: S5 Table — (DOC) [file pgen.1005368.s015.doc]

###### Table S5: PCR Primers used to analyze the targeted *Rfx2* locus.

| amplicon | primers | | sequence (5’-3’) | size (kb) |
| --- | --- | --- | --- | --- |
| targeted insertion  short arm | 1 | SK 1534 F | TCAGCCTGCTCTCTTATATC | 2.6 |
|  | 2 | SK 1535 R | TATGCTATACGAAGTTATAAGC |  |
| targeted insertion  long arm | 3 | SK 1540 F | GAACTTCAGAGCGCTTTTGA | 5.8 |
|  | 4 | SK 1541 R | GGAAGCCTCAGTGGATGGTA |  |
| wild type locus | 5 | SK 1561 F | TCATGACTTCCAGCTGTCCA | 0.3 |
|  | 6 | SK 1557 R | GGCTAGCCTGGGCTATATGA |  |
| deleted *Rfx2* | 7 | F-JT309 (F-genoRfx2) | AGAATCTGCCCCTTGGCTAT | 0.9 |
|  | 8 | R-JT310 (R-genoRfx2) | TGTCACCCACCTAGGCTTCT |  |
| floxed *Rfx2* | 7 | F-JT309 (F-genoRfx2) | AGAATCTGCCCCTTGGCTAT | 1.1 |
|  | 9 | SK 1536 R | CTCGAGGGAGCTTCAAAAG |  |
| *Rfx2* ex7-ex8 | 10 | SK 1271 F (ex 7) | CCAGTGGCTTCTGGACAATTACGA | 0.3 |
|  | 11 | SK 1274 R (ex 8) | TAGGCTTCTGGTGTGTGGGTTGCT |  |
| *Atp5a1* | 12 | SK 1196 (ex 10) | GGAGCCCAGCAAGATCACAAAGTT | 0.2 |
|  | 13 | SK 1197 (ex 11) | ATCTGGTGACAGTGACAGGGCTTT |  |
