# Supplementary material for: RFX2 Is a Major Transcriptional Regulator of Spermiogenesis
Source: PLoS Genet. 2015 Jul 10;11(7):e1005368. doi: 10.1371/journal.pgen.1005368 (PMC4498915; doi:10.1371/journal.pgen.1005368)
Supplement: S1 Text — (DOCX) [file pgen.1005368.s016.docx]

**SUPPLEMENTARY METHODS**

**Library preparation, sequencing and read mapping to the reference genome**

RNA was isolated from decapsulated testes using an RNeasy kit (Qiagen, Valencia, CA). RNA were preparated from 5 WT and 4 *Rfx2*^-/-^ mice at P30 and 5 WT and 6 *Rfx2-/-* mice at P21. cDNA libraries were constructed by the Genomics Platform of the University of Geneva using the Illumina TruSeq RNA Sample Preparation Kit according to the manufacturer's protocol. Libraries were sequenced using single reads (46nt-long) on Illumina HiSeq2000. FastQ reads were mapped to the ENSEMBL reference genome (GRCm38.70) using Bowtie [5] with standard settings, except that any reads mapping to more than one location in the genome (ambiguous reads) were discarded (m = 1). Then a "pileup" format file containing the count of reads per base aligned to each location across the length of the genome was generated using SAMTools [6]. Sequence data has been submitted to the GEO database under the accession number GSE63130

(http://www.ncbi.nlm.nih.gov/geo/query/acc.cgi?acc=GSE63130).

**Unique gene model construction and gene coverage reporting**

A unique gene model was used to quantify reads per gene. Briefly, the model considers all annotated exons of all annotated protein coding isoforms of a gene to create a unique gene where the genomic region of all exons are considered coming from the same RNA molecule and merged together.

**RNAseq analysis**

RNA was isolated from decapsulated testes using an RNeasy kit (Qiagen, Valencia, CA). cDNA libraries were constructed by the Genomics Platform of the University of Geneva using the Illumina TruSeq RNA Sample Preparation Kit according to the manufacturer's protocol. Libraries were sequenced using single reads (50nt-long) on Illumina HiSeq2000. FastQ reads were mapped to the ENSEMBL reference genome (GRCm38.70) using STAR [7] with standard settings, except that any reads mapping to more than one location in the genome (ambiguous reads) were discarded (m = 1). All reads reporting the exons of each unique gene model were reported using featureCounts [8], excluding the reads overlapping exons from different genes (ambiguous origin). Library size normalizations and differential gene expression calculations were performed using the package DESeq [9] designed for the R software [10]. 5 and 6 biological replicates were used for the *Rfx2+/+* and the *Rfx2-/-* mice respectively at P21, while 5 and 4 biological replicates were used for the *Rfx2+/+* and the *Rfx2-/-* mice respectively at P30. Only genes having a significant fold-change (p-value ≤ 0.01) were considered for the rest of the RNAseq analysis. Sequence data has been submitted to the GEO database under the accession number GSE63130 (http://www.ncbi.nlm.nih.gov/geo/query/acc.cgi?acc=GSE63130).

- - 1. ChIPseq analysis

Chromatin immunoprecipitation was performed using anti-RFX2 antibody (Santa-Cruz, antibody C-15: sc-10657) as described [11]. Immunoprecipitated DNA was sequenced using the Illumina HiSeq 2000 platform. Two replicates were done, as proposed by the ENCODE consortium [12]. Inputs were used as control. The library for duplicate 2 was sequenced twice in order to obtain more reads. The second sequencing of the library for duplicate 2 was contaminated with human DNA sequences so reads were first mapped on human genome (GRCh38.76) using Bowtie 0.12.7 [5] with standard settings in order to remove reads from human origin. Reads that did not map on human genome were pooled with reads obtained from the first sequencing of replicate 2. Reads were mapped to the mouse genome (GRCm38.70) using Bowtie 0.12.7 [5] with standard settings, except that any reads mapping to more than one location in the genome (ambiguous reads) were discarded (m = 1). Fragment length was estimated using cross-correlation [12]. Peak calling was first done for each replicate using MACS2 [13] with the default settings, except that the “--no-model” setting was used and the shift size parameter was set to half of the estimated fragment length, in order to get an estimation of the number of discovered peaks in each replicate. Peaks were next called using MACS2 [13] with “--no-model” and “--to-large” settings, p=0.01 as threshold and shift-size parameter set to half of the estimated fragment length, using input as control. Number of reported peaks was limited to five time the number of peaks discovered in the first peak calling, in order to assess the Irreproducible Discovery Rate (IDR) between both replicates, based on replicability, not primarily the peak caller's statistics. Reproducible peaks were obtained by assessing the IDR between both replicates using a threshold of 0.01 [12]. 2716 peaks were considered reproducible. Peaks overlapping by at least 1nt with unique gene model promoters (-1000bp to +500bp of each unique gene model Transcription Starting Site) were considered as promoter located. The rest of the peaks overlapping (at least 1nt) with any unique gene model exons were classified as exon located. The rest of the peaks overlapping (at least 1nt) with any unique gene model introns were classified as intron located. Other peaks were considered as intergenic. Genome coverage was obtained using the genomeCoverageBed function from Bedtools [14]. Coverages were expressed in RPM (Reads Per Million) of mapped reads. Sequence data has been submitted to the GEO database under the accession number GSE63130 (http://www.ncbi.nlm.nih.gov/geo/query/acc.cgi?acc=GSE63130).

***De novo* motif discovery and motif enrichment analysis**

Promoter sequences (-500bp to +50bp relative to the TSS) were extracted, oriented according to gene orientation and used as input for de novo motif discovery using the cosmo package [15] designed for R [10]. The probabilistic model used for the motif discovery was the zero-or-one-occurrence-per-sequence (ZOOPS), considering only the orientation of each promoter and motif lengths between 10nt and 20nt. Pscan [16] was used to identify potential TF-binding-sites (TFBSs) that are overrepresented in each promoter category, using the JASPAR database [1] as matrices. TFBSs with pvalues lower than 0.001 were considered to be significantly overrepresented. Peaks sequences were ordered by pvalue (from the lowest to the highest), and only up to the 100 best sequences were kept for motif discovery analysis. The probabilistic model used for the motif discovery was the zero-or-one-occurrence-per-sequence (ZOOPS), using the sequences surrounding each peak as background sequences and motif lengths between 10nt and 20nt.

**Gene Ontology**

GO term enrichment was performed using either the DAVID server [17], GOEAST [18] or AmiGO [19]. Table S4 and Figure S7, 8, list GO terms by increasing p value <0.1 (with Bonferonni’s correction) obtained from AmiGO.

**Establishment of developmental expression profiles**.

Data from Laiho et al 2013 [4] was used to generate expression patterns during WT spermatogenesis for genes that are downregulated in *Rfx2^-/-^* testis or *Crem^-/-^* testis. Only genes having a read coverage greater than 5 RPKM for at least 1 time point in their data set were considered for analysis. Data was converted to expression relative to maximum for each gene. To identify genes exhibiting expression profiles consistent with activation by RFX2 or CREM, only genes for which the expression data could be fitted to a sigmoid curve having an inflection point at a time point greater than 10 days were retained.

**Supplemental references**

1. Sandelin A, Alkema W, Engstrom P, Wasserman WW, Lenhard B (2004) JASPAR: an open-access database for eukaryotic transcription factor binding profiles. Nucleic Acids Res 32: D91-94.

2. Kosir R, Juvan P, Perse M, Budefeld T, Majdic G, et al. (2012) Novel Insights into the Downstream Pathways and Targets Controlled by Transcription Factors CREM in the Testis. PLoS ONE 7: e31798.

3. Martianov I, Choukrallah MA, Krebs A, Ye T, Legras S, et al. (2010) Cell-specific occupancy of an extended repertoire of CREM and CREB binding loci in male germ cells. BMC Genomics 11: 530.

4. Laiho A, Kotaja N, Gyenesei A, Sironen A (2013) Transcriptome profiling of the murine testis during the first wave of spermatogenesis. PLoS ONE 8: e61558.

5. Langmead B, Trapnell C, Pop M, Salzberg SL (2009) Ultrafast and memory-efficient alignment of short DNA sequences to the human genome. Genome Biol 10: R25.

6. Li H, Handsaker B, Wysoker A, Fennell T, Ruan J, et al. (2009) The Sequence Alignment/Map format and SAMtools. Bioinformatics 25: 2078-2079.

7. Dobin A, Davis CA, Schlesinger F, Drenkow J, Zaleski C, et al. (2013) STAR: ultrafast universal RNA-seq aligner. Bioinformatics 29: 15-21.

8. Liao Y, Smyth GK, Shi W (2014) featureCounts: an efficient general purpose program for assigning sequence reads to genomic features. Bioinformatics 30: 923-930.

9. Anders S, Huber W (2010) Differential expression analysis for sequence count data. Genome Biol 11: R106.

10. Team RDC (2011) R: a language and environment for statistical computing. R Foundation for Statistical Computing, Saturday, Jan 01, 2011.

11. Staehli F, Ludigs K, Heinz LX, Seguin-Estevez Q, Ferrero I, et al. (2012) NLRC5 deficiency selectively impairs MHC class I- dependent lymphocyte killing by cytotoxic T cells. J Immunol 188: 3820-3828.

12. Landt SG, Marinov GK, Kundaje A, Kheradpour P, Pauli F, et al. (2012) ChIP-seq guidelines and practices of the ENCODE and modENCODE consortia. Genome Res 22: 1813-1831.

13. Liu T (2014) Use model-based Analysis of ChIP-Seq (MACS) to analyze short reads generated by sequencing protein-DNA interactions in embryonic stem cells. Methods Mol Biol 1150: 81-95.

14. Quinlan AR, Hall IM (2010) BEDTools: a flexible suite of utilities for comparing genomic features. Bioinformatics 26: 841-842.

15. Bembom O, Keles S, van der Laan MJ (2007) Supervised detection of conserved motifs in DNA sequences with cosmo. Stat Appl Genet Mol Biol 6: Article8.

16. Zambelli F, Pesole G, Pavesi G (2009) Pscan: finding over-represented transcription factor binding site motifs in sequences from co-regulated or co-expressed genes. Nucleic Acids Res 37: W247-252.

17. Huang da W, Sherman BT, Lempicki RA (2009) Bioinformatics enrichment tools: paths toward the comprehensive functional analysis of large gene lists. Nucleic Acids Res 37: 1-13.

18. Zheng Q, Wang XJ (2008) GOEAST: a web-based software toolkit for Gene Ontology enrichment analysis. Nucleic Acids Res 36: W358-363.

19. Ashburner M, Ball CA, Blake JA, Botstein D, Butler H, et al. (2000) Gene ontology: tool for the unification of biology. The Gene Ontology Consortium. Nat Genet 25: 25-29.
